# A generative model for constructing nucleic acid sequences binding to a protein

**DOI:** 10.1186/s12864-019-6299-4

**Published:** 2019-12-27

**Authors:** Jinho Im, Byungkyu Park, Kyungsook Han

**Affiliations:** Department of Computer Engineering, Inha University, Incheon, 22212 South Korea

**Keywords:** Aptamer, Protein-nucleic acid binding, Recurrent neural network

## Abstract

**Background:**

Interactions between protein and nucleic acid molecules are essential to a variety of cellular processes. A large amount of interaction data generated by high-throughput technologies have triggered the development of several computational methods either to predict binding sites in a sequence or to determine whether a pair of sequences interacts or not. Most of these methods treat the problem of the interaction of nucleic acids with proteins as a classification problem rather than a generation problem.

**Results:**

We developed a generative model for constructing single-stranded nucleic acids binding to a target protein using a long short-term memory (LSTM) neural network. Experimental results of the generative model are promising in the sense that DNA and RNA sequences generated by the model for several target proteins show high specificity and that motifs present in the generated sequences are similar to known protein-binding motifs.

**Conclusions:**

Although these are preliminary results of our ongoing research, our approach can be used to generate nucleic acid sequences binding to a target protein. In particular, it will help design efficient in vitro experiments by constructing an initial pool of potential aptamers that bind to a target protein with high affinity and specificity.

## Introduction

Due to recent advances in high-throughput experimental technologies, a large amount of data on interactions between proteins and nucleic acids have been generated. Motivated by the increased amount of data on protein-nucleic acid interactions, several machine learning methods have been used either to predict binding sites in a sequence [1–4] or to determine if an interaction exists between a pair of sequences [5–9].

Among the machine learning methods, variants of neural networks were applied to predict the interactions between proteins and nucleic acids. For example, DeepBind [5] is a convolutional neural network trained on a huge amount of data from high-throughput experimental technologies. For the problem of predicting protein-binding sites of nucleic acid sequences, DeepBind contains hundreds of distinct prediction models, each for a different target protein. As output, it provides a predictive binding score without suggesting protein-binding sites in the input nucleic acid sequence. Nonetheless, it provides informative predictions for many target proteins, so we used DeepBind to estimate the affinity and specificity of nucleic acid sequences generated by our model for a target protein.

A more recent model called DeeperBind [10] predicts the protein-binding specificity of DNA sequences using a long short-term recurrent convolutional network. By employing more complex and deeper layers, DeeperBind showed a better performance than DeepBind for some proteins, but its use is limited to the datasets from protein-binding microarrays. Both DeepBind and DeeperBind are classification models rather generative models, so cannot be used to construct nucleic acid sequences that potentially bind to a target protein.

There are a few computational methods that generate protein-binding nucleic acid sequences. Most of them include two steps: generating candidate sequences and testing the sequences. For instance, Kim et al. [11] generated a large number of RNA sequences using nucleotide transition probability matrices and selected candidate sequences with specified secondary structures and motifs. Their approach is quite exhaustive and requires a large amount of computational power. Zhou et al. [12] generated RNA sequences that can form a desired RNA motif, and selected potent aptamers by molecular dynamics simulation-based virtual screening. Hoinka et al. [13] developed a program called AptaSim for simulating the selection dynamics of HT-SELEX experiments based on a Markov model.

The main difference of our approach from the others is that our approach is a deep learning model that can be trained directly on data from high-throughput experiments such as HT-SELEX or CLIP-seq. After being trained on experimental data, our model generates sequences similar to those in a training dataset, and evaluates the sequences with respect to binding affinity and specificity to a target protein. A limitation of our model is that it requires experimental data for training and a classifier of protein-binding nucleic acids. However, this limitation is expected to be overcome in the near future as a large amount of experimental data is being generated through high-throughput experiments.

This paper presents a generative model that constructs potential aptamers for a target protein. Aptamers are synthetic but biologically active, short single-stranded nucleic molecules which bind to a target molecule with high affinity and specificity [14]. The preliminary results show that our approach can generate nucleic acid sequences that bind to a target protein with high affinity and specificity, which will definitely help design in vitro or in vivo experiments to finalize aptamers for target proteins. To the best of our knowledge, this is the first attempt to generate potential aptamers using a recurrent neural network.

## Materials and methods

### Data set

The data set used for training the generator model was obtained from the DeepBind site at http://tools.genes.toronto.edu/deepbind/nbtcode. The data set includes a large number DNA sequences binding to one of 396 transcription factors (TFs). In the data set, 20-mer DNA sequences bind to most TFs (320 out of 396 TFs), 14-mer DNA sequences bind to 14 TFs, and 40-mer DNA sequences bind to 25 TFs. Thus, we selected the most typical length of 20 as the length of DNA sequences generated by our model.

In the data set, setA contains positive data (i.e., protein-binding DNA sequences) and setB contains negative data (i.e., non-binding DNA sequences). We used setA to train our generator model. For comparison of our method with others, the HT-SELEX data was obtained from https://www.ncbi.nlm.nih.gov/bioproject/371436. Both data sets are also available in Additional file 1.

### Sequence generator

A recurrent neural network (RNN) is capable of learning the property of sequential data such as time series data or text data. However, RNN suffers from the vanishing gradient problem, in which the gradients vanish and consequentially the parameters are not updated during back propagation. Long short-term memory (LSTM) solves the vanishing gradient problem of RNN by introducing a gating mechanism [15]. LSTM allows the network to determine when and what to remember or forget. LSTM has shown a great performance in speech recognition [16] and language translation [17].

**Fig. 1:**
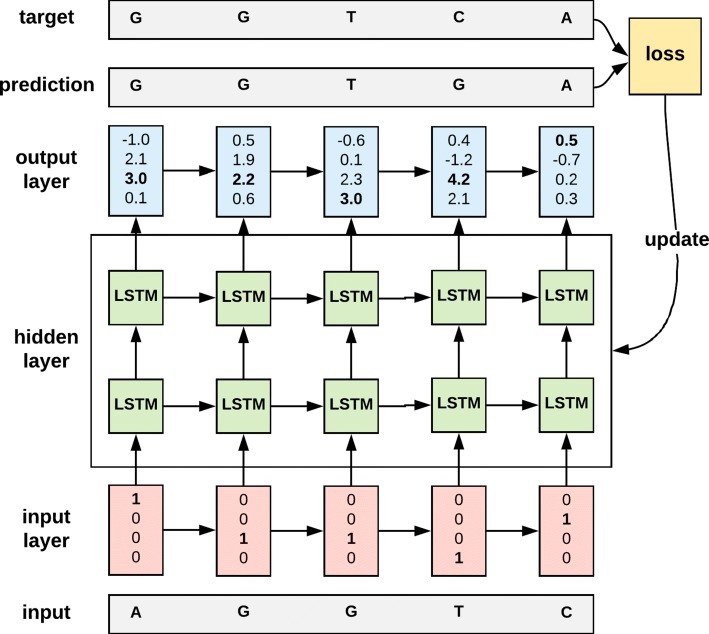
The architecture of the sequence generator. The loss is used to update the hidden neurons in the hidden layer using the RMSProp algorithm [18]

We implemented a generator model of nucleic acid sequences using char-rnn (https://github.com/karpathy/char-rnn). Our model is composed of two layers of LSTM with 128 hidden neurons (Fig. 1). Given a sequence of characters, it reads one character of the sequence at a time and predicts the next in the sequence.

In the LSTM model, the batch size (B) specifies how many streams of data are processed in parallel at one time. The sequence length (S) specifies the length of each stream (S=20 in our dataset). Suppose that an input file to a model has k DNA sequences of 20 nucleotides and that *N*=*k*×20. Then, the input file of N characters is split into data chunks of size *B*×20. By default, 95% of the data chunks are used for training and 5% of the chunks are used to estimate the validation loss. The input file is split into data chunks and fed to the LSTM layers with default settings. In our study, we used the default value of 50 for the batch size (B).

The LSTM model was trained in the following way (Eq. 1). Let *x*_*t*_ be a vector representing the *t*-th nucleotide in the input sequence. Only one element of *x*_*t*_ is 1 and the others are 0. *y*_*t*_ is a class indicator of *n*_*t*_ defined by Eq. 1. The LSTM calculates *z*_*t*_ for *x*_*t*_ (Eq. 2). Softmax changes *z*_*t*_ to a vector of values between 0 and 1 that sum to 1, and *softmax*_*j*_ is the *j*-th element of the output of the softmax (Eq. 3). The loss is the mean of the negative log-likelihood of the prediction (Eq. 4). The loss is used to update the hidden neurons in the hidden layer using the RMSProp algorithm [18]. When generating a sequence, the model takes a vector (0.25,0.25,0.25,0.25) as *x*_1_ and computes *softmax*(*z*_*t*_), a multinomial distribution of nucleotides. One character is sampled from the distribution and the vector of the character fed back to the model as *x*_2_. This process is repeated until it reaches the pre-determined length of the sequence.

1 $$\begin{array}{*{20}l} &x_{t} = \text{4-bit number representing a nucleotide\;} \\&\qquad \, n_{t} \in \{A, C, G, T (U) \} \\ &y_{t} = \left\{\begin{array}{ll} 1 & \text{if} \; n_{t} = A \\ 2 & \text{if} \; n_{t} = C \\ 3 & \text{if} \; n_{t} = G \\ 4 & \text{if} \; n_{t} = T (U)\\ \end{array}\right. \end{array} $$

2 $$\begin{array}{*{20}l} z_{t} = LSTM(x_{t}) \end{array} $$

3 $$\begin{array}{*{20}l} softmax_{j}(z_{t})=e^{z_{tj}}/\textstyle\sum_{k=1}^{4}e^{z_{tk}}, j \in \{1, 2, 3, 4\} \end{array} $$

4 $$\begin{array}{*{20}l} loss=-\sum_{t=1}^{\vert x\vert}ln(softmax_{y_{t}}(z_{t})) / \vert x\vert \end{array} $$

For protein-binding DNA sequences, the model was trained on a set of DNA sequences, which were identified by HT-SELEX experiments as binding sequences to human transcription factors [19]. Among the transcription factors, we selected those with a known aptamer. Since the DNA sequences used in training the model were 20 nucleotide long, the length of nucleic acid sequences generated by the model was also set to 20 nucleotides. When training the model, the results were evaluated with respect to two measures: loss and intersection to union (IU) ratio, which are defined by Eqs. 4 and 5, respectively.

5 $$ \text{IU}\, \text{ratio} = \frac{\{\text{training} \; \text{sequences}\} \cap \{\text{generated} \; \text{sequences}\}}{\{\text{training} \; \text{sequences}\} \cup \{\text{generated} \; \text{sequences}\}}  $$

**Fig. 2:**
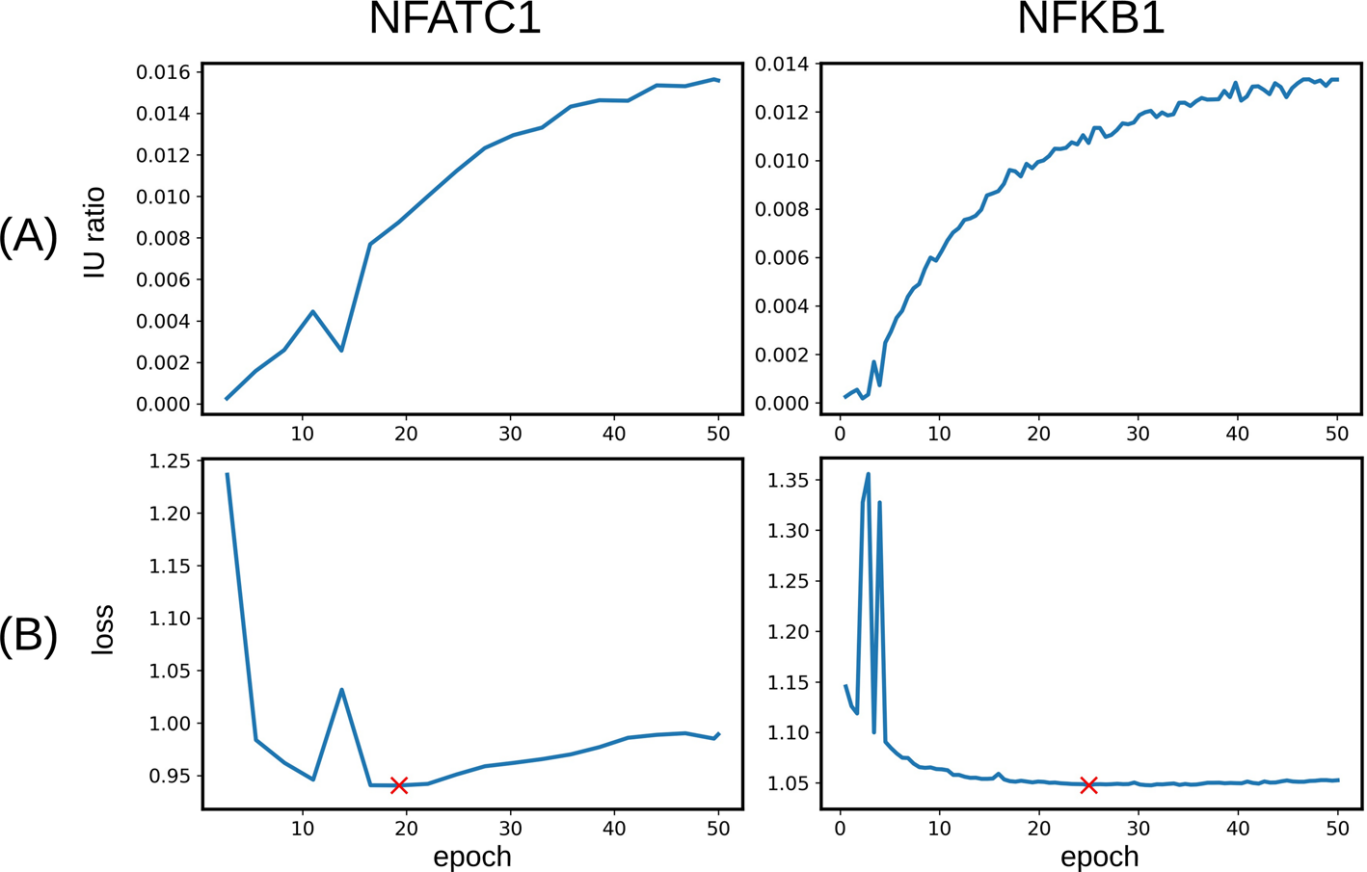
**a** The IU ratio of the model during the first 50 epochs of training for NFATC1 and NFKB1. **b** The loss of the model during the first 50 epochs of training. The red symbol ’x’ represents the minimum loss point

Figure 2 shows the IU ratios and loss values of the model during the first 50 epochs of training for NFATC1 and NFKB1. For both NFATC1 and NFKB1, the IU ratio was increased as the model was trained longer (Fig. 2a). In contrast to the IU ratio, the loss tended to be decreased after a certain point as the model was trained longer, but the decreasing trend was not monotonic. The loss of the model for NFKB1 converged to ∼1.05, whereas that for NFATC1 was increased slightly after reaching to the minimum loss of 0.95 at epoch 19.

The model with the maximum IU ratio generated many redundant sequences. About 25% and 33% of the sequences generated by the model for NFKB1 and NFATC1 were duplicated sequences, respectively. Thus, we selected a generator model with the minimum loss value rather than one with the maximum IU ratio to construct various sequences which are similar, but not exactly the same, to those in the training set.

### Binding affinity and specificity

**Fig. 3:**
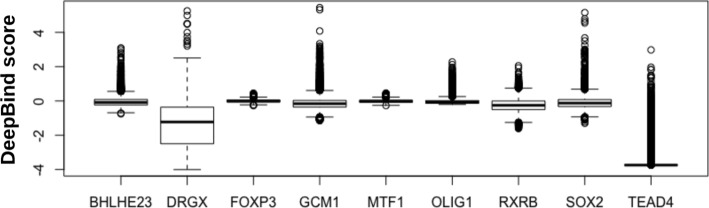
DeepBind scores of random sequences, calculated by 9 DeepBind models for 9 proteins (BHLHE23, DRGX, FOXP3, GCM1, MTF1, OLIG1, RXRB, SOX2, and TEAD4)

To evaluate the binding affinity and specificity of nucleic acid sequences to a target protein, we used the predictive binding score of DeepBind (hereafter called DeepBind score) [5]. Figure 3 shows DeepBind scores of random sequences in 10 DeepBind models. As shown in Fig. 3, the scale of DeepBind scores is arbitrary, thus DeepBind scores from different DeepBind models are not directly comparable.

**Fig. 4:**
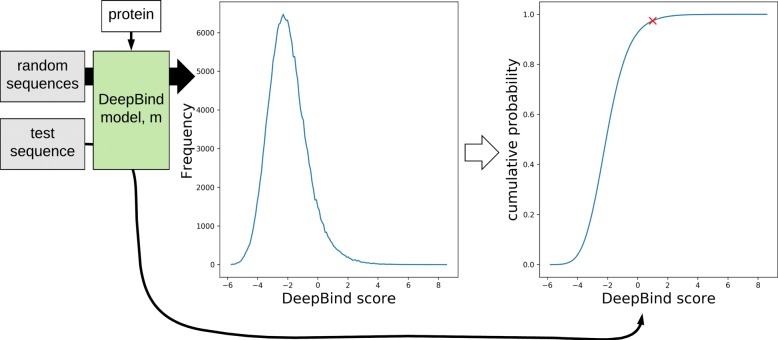
The procedure for computing the binding affinity of a sequence *s* to a target protein *p*. After computing DeepBind scores of 200,000 random sequences by a DeepBind model *m* for *p*, an empirical cumulative distribution function was derived from the DeepBind scores. The function is discrete, but seems continuous due to a large number of data points. The binding affinity of *s* to *p* is the probability that the DeepBind score of *s* would be higher than that of a random sequence

To make DeepBind scores comparable, we defined the binding affinity (*AF*) of a nucleic acid sequence *s* to a target protein *p* as the probability that the DeepBind score of *s* would be higher than that of a random sequence. To obtain an approximate value of the probability, we ran DeepBind on 200,000 random DNA sequences of 20 nucleotides and computed their binding affinity by Eq. 6. Since the binding affinity is a probability, it is always in the range of [0, 1]. In the equation, *Score*_*m*_(*s*) and *Score*_*m*_(*r*_*i*_) represent the score of a sequence *s* and the score of the *i*-th random sequence, respectively, computed by DeepBind model *m*. The procedure for computing the binding affinity is illustrated in Fig. 4.

6 $$ \begin{array}{l} AF_{p}(s) = \frac{1}{n}\sum_{i=1}^{n}\delta(Score_{m}(s) \geq Score_{m}(r_{i})), \\ \\ \text{where} \; \delta(A)= \text{1 if an event A occurs;} \; \delta(A)= \text{0 otherwise}. \end{array}  $$

Table 1 shows some positive data used for training and testing DeepBind models for several target proteins along with AUC values in testing. Different DeepBind models show very different AUC values, ranging from 0.499 for FOXP3 to 0.990 for TEAD4. The AUC value of 0.499 in testing is close to random guessing.

**Table 1:** Part of positive data from [19] used for training and testing DeepBind

Protein	Type	Species	Family	Experiment	AUC in test data
TEAD4	TF	*H. sapiens*	TEA	ChIP-seq	0.990
NFATC1	TF	*H. sapiens*	Rel	SELEX	0.909
DRGX	TF	*H. sapiens*	Homeodomain	SELEX	0.897
GCM1	TF	*H. sapiens*	GCM	SELEX	0.841
NFKB1	TF	*H. sapiens*	Rel	SELEX	0.771
OLIG1	TF	*H. sapiens*	bHLH	SELEX	0.733
RXRB	TF	*H. sapiens*	Nuclear receptor	SELEX	0.720
SOX2	TF	*H. sapiens*	Sox	SELEX	0.605
BHLHE23	TF	*H. sapiens*	bHLH	SELEX	0.557
MTF1	TF	*H. sapiens*	C2H2 ZF	SELEX	0.538
FOXP3	TF	*H. sapiens*	Forkhead	SELEX	0.499
MBNL1	RBP	*H. sapiens*	Znf	RNAcompete	–

Negative data was prepared by shuffling dinucleotides. TF: transcription factor. RBP: RNA binding protein. AUC of DeepBind for MBNL1 is not available

We defined the binding specificity (*SP*) of a nucleic acid sequence *s* to a target protein *p* by Eq. 7. The binding specificity of *s* to *p* is the difference between the AUC-weighted binding affinity *AF* of *s* to *p* and the AUC-weighted mean *AF* of *s* to all other proteins except *p*. In the equation, *M* is a set of all generator models trained on data from the same type of experiment as *m*. The binding affinity *AF* is weighted by AUC to reflect the reliability of each model. When the AUC value is not available, *AF* is not weighted by AUC (i.e., *AUC*_*m*_ = 1 for every model *m*).

7 $$ \begin{array}{l} M^{c}\;=\;M\;-\;\{m\} \\\\ SP_{p}(s)\;=\;AF_{p}(s)\cdot AUC_{m}-\frac1{\vert M^{c}\vert}\sum_{k \in M^{c}}AF_{k}(s)\cdot AUC_{k} \end{array}  $$

### Algorithm

To construct potential aptamers for a protein target, our model requires three inputs: a target protein, a training set of nucleic acid sequences binding to the target protein, and a set of DeepBind models. A DeepBind model for the target protein should be included in the input. After training the model on the training dataset for 50 epochs, we select a model with the lowest loss value. The selected model is used to generate nucleic acid sequences, and the binding affinity and specificity of the generated sequences to the target protein are computed using Eq. 3 and 4, and the top 100 sequences with the highest binding specificity are chosen as potential aptamers of the target protein. A high-level description of our approach is outlined in Algorithm 1.


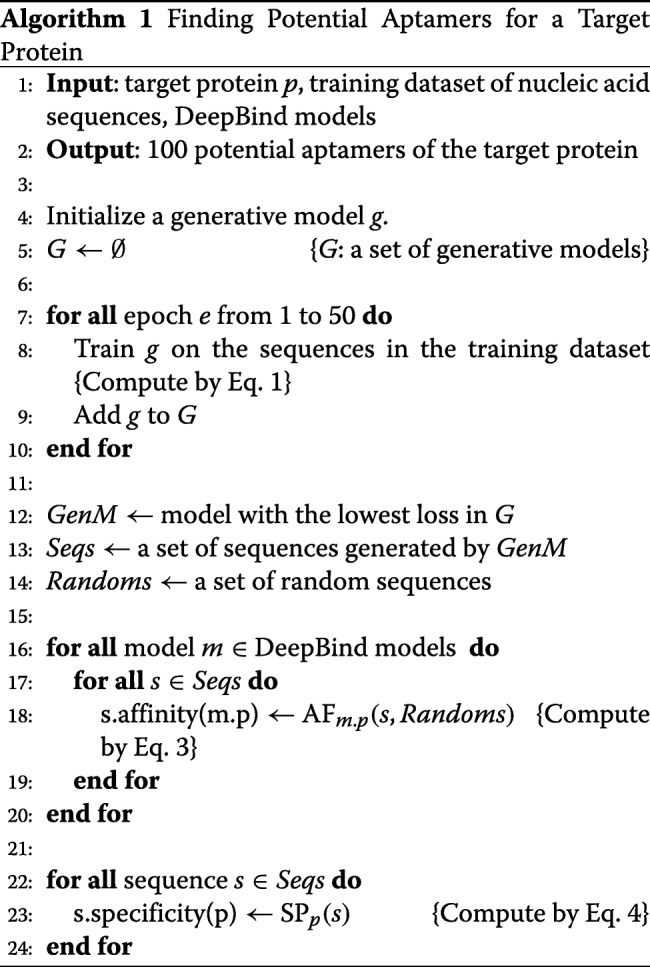


## Results and discussion

### Binding affinity of generated sequences

To examine the protein-binding affinity of DNA sequences, we generated DNA sequences binding to several proteins shown in Table 1. For each target protein, Table 2 shows AUC of the protein’s DeepBind model and median protein-binding affinity *AF* of the generated sequences and random sequences. For comparison we used the median *AF* value instead of the mean *AF* because outliers can distort the mean. As shown in Table 2, the median *AF* values were proportional to the AUC values of DeepBind models. The sequences generated by our model showed a much higher median *AF* than random sequences, except for SOX2.

**Table 2:** The median binding affinity *AF* of the generated sequences and random sequences to target protein *p* with AUC of DeepBind model of *p*

Protein	DRGX	GCM1	OLIG1	RXRB	SOX2	BHLHE23	MTF1	FOXP3
AUC	0.897	0.841	0.733	0.720	0.605	0.557	0.538	0.499
Median *AF* (Generated)	0.999	0.995	0.763	0.644	0.473	0.513	0.557	0.502
Median *AF* (Random)	0.497	0.500	0.504	0.494	0.504	0.501	0.502	0.501

**Fig. 5:**
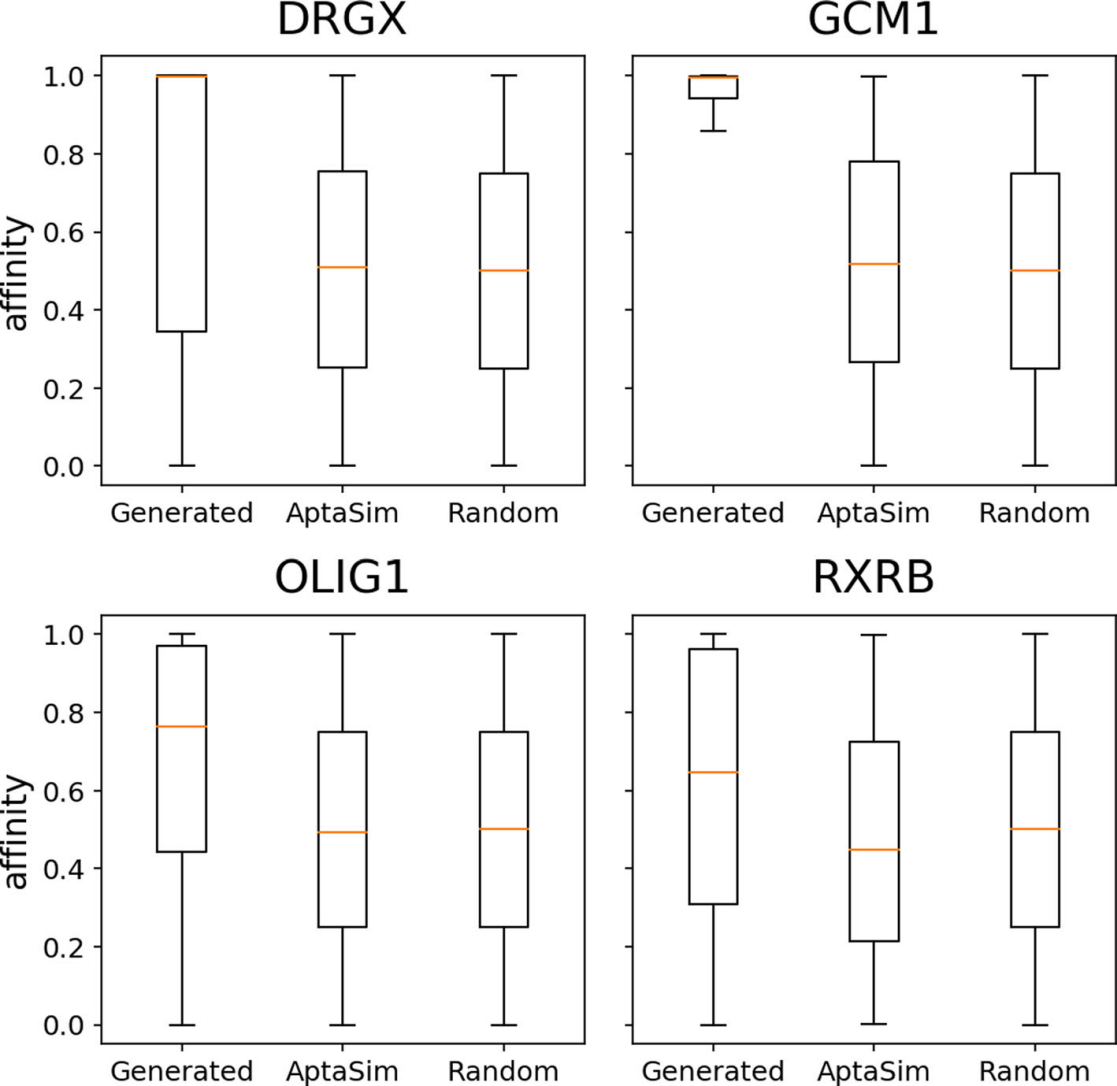
The binding affinity *AF* of the nucleic acid sequences generated by our model, AptaSim, and random generator for four target proteins (DRGX, GCM1, OLIG1 and RXRB)

For comparison of our model with AptaSim, we downloaded the HT-SELEX data [19] and ran AptaSim in the AptaSuite collection [20]. The sequences in the first SELEX round of target proteins were used as input to AptaSim. Figure 5 shows the distribution of *AFs* of the sequences generated by our model, AptaSim and random generator for four target proteins (DRGX, GCM1, OLIG1 and RXRB). The sequences generated by AptaSim showed similar binding affinity as random sequences, but both showed much lower binding affinity than the sequences generated by our model. The nucleic acid sequences used for comparison are available in Additional file 2.

### Protein-binding dNA sequence motif

**Fig. 6:**
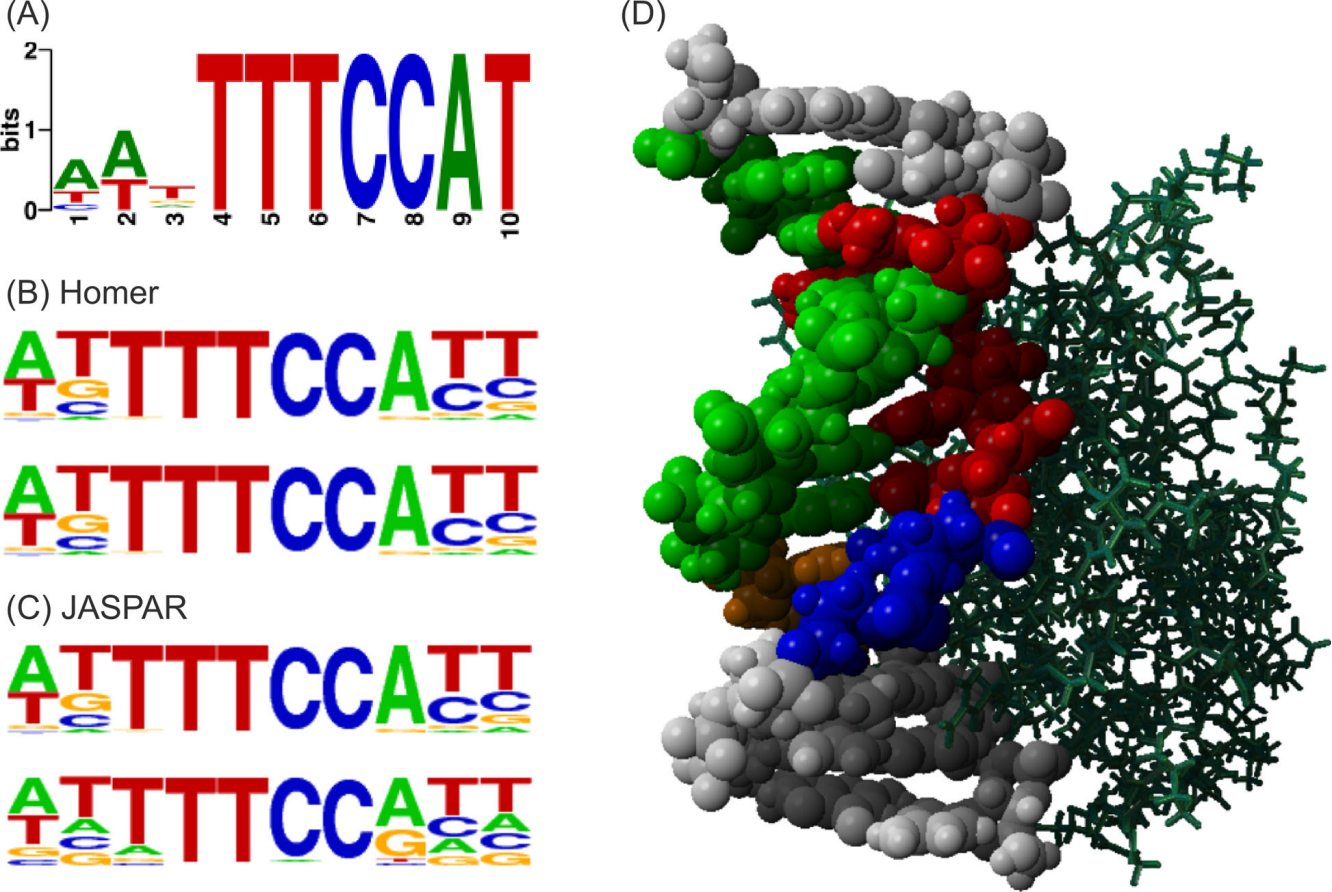
**a** Sequence motif conserved in the DNA sequences generated by our model as binding sequences of NFATC1. **b** Known NFATC1-binding DNA motifs in Homer [22]. **c** Known NFATC1-binding DNA motifs in JASPAR [23]. **d** Structure of a complex of NFATC1 and DNA (PDB ID: 1A66)

We generated about 200,000 DNA sequences for NFATC1 using our model, and found a motif (shown in Fig. 6a) conserved in the DNA sequences using DREME [21]. The motif found in the generated DNA sequences was also corroborated by a protein-DNA complex in PDB (Fig. 6d) and known motifs (Figs. 6b and c) from the Homer [22] and JASPAR [23] databases.

**Fig. 7:**
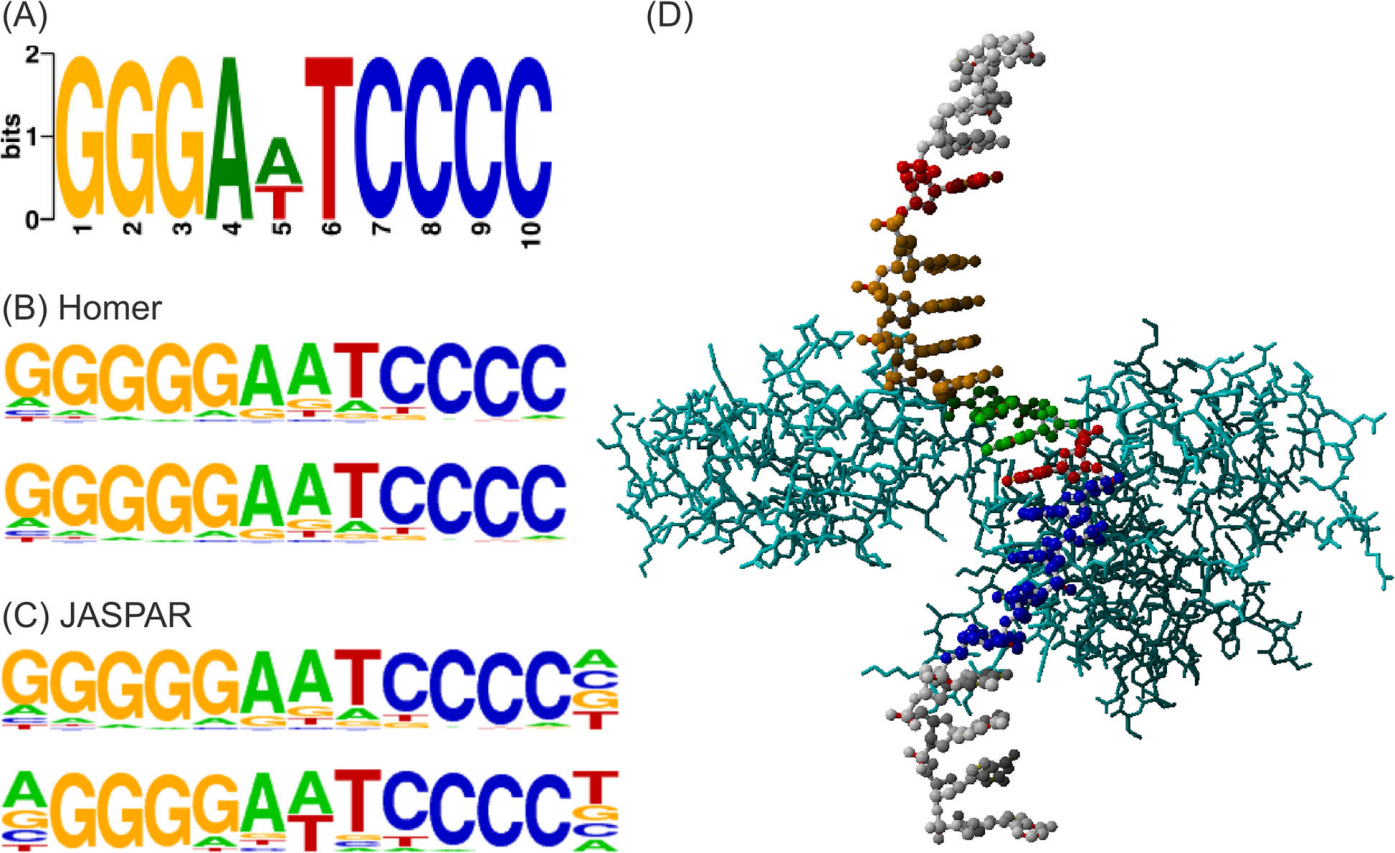
**a** Sequence motif conserved in the DNA sequences generated by our model as binding sequences of NFKB1. **b** Known NFKB1-binding DNA motifs in Homer [22]. **c** Known NFKB1-binding DNA motifs in JASPAR [23]. **d** Structure of a complex of NF-kappa B and DNA (PDB ID: 1SVC)

In a similar way, we obtained a sequence motif conserved in the DNA sequences for NFKB1 (Fig. 7). DNA sequences and their binding specificity for NFATC1 and NFKB1 are available in Additional file 3.

### Comparison with known aptamers

For comparative purposes of the sequences generated by our model to known aptamers, we selected top 100 DNA sequences with a high binding specificity. We aligned the sequences to each of the known aptamers for NFATC1 [24] and NFKB1 [25] (Additional file 4) using the EMBOSS needleman [26].

**Fig. 8:**
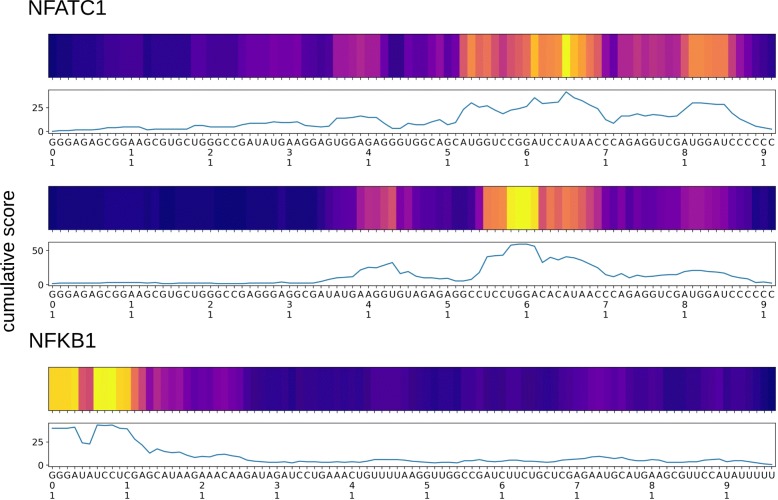
The alignment of the generated DNA sequences to the known NFATC1-binding aptamer [24] (top) and NFKB1-binding aptamer [25] (bottom). Top 100 sequences with high binding specificity scores were selected for the alignment. The cumulative binding specificity score of the aligned sequences is shown in the line chart and heatmap. In the heatmap, a position with a high accumulated binding specificity is shown in yellow and that with a low binding specificity is shown in navy

As shown in Fig. 8, two alignments of DNA sequences to the NFATC1 aptamer revealed a similar pattern of binding specificity. In the first alignment of DNA sequences to the NFATC1 aptamer, the highest accumulated score of the binding specificity was observed right after the 40-mer region in 5^′^-GGGAGAGCGGAAGCGUGCUGGGCC-N40-CAUAACCCAGAGGUCGAUG GAUCCCCCC- 3^′^. But, in the second alignment the highest score was found in the 40-mer region. These results imply that our approach is useful in finding potential aptamers binding to a target protein. In the alignment, the highest score was observed in the 5^′^ end of the aptamer, which is a primer site of a random library used when selecting the aptamer.

We used the model to generate protein-binding RNA sequences as well. As we did for DNA sequences, we trained the model on MBNL1-binding RNA sequences from CLIPdb [27], which were identified by CLIP-seq experiments. We selected top 100 RNA sequences with a high binding specificity (Additional file 3), and aligned them to known MBNL1-binding aptamers [28] (Additional file 4). The known aptamers contain 32-mer MBNL1-binding regions, which are flanked by two constant regions (5^′^-GGGAAUGGAUCCACAUCUACGAAUUC-N32-AAGACUCGAUACGUGACGA ACCU- 3^′^).

**Fig. 9:**
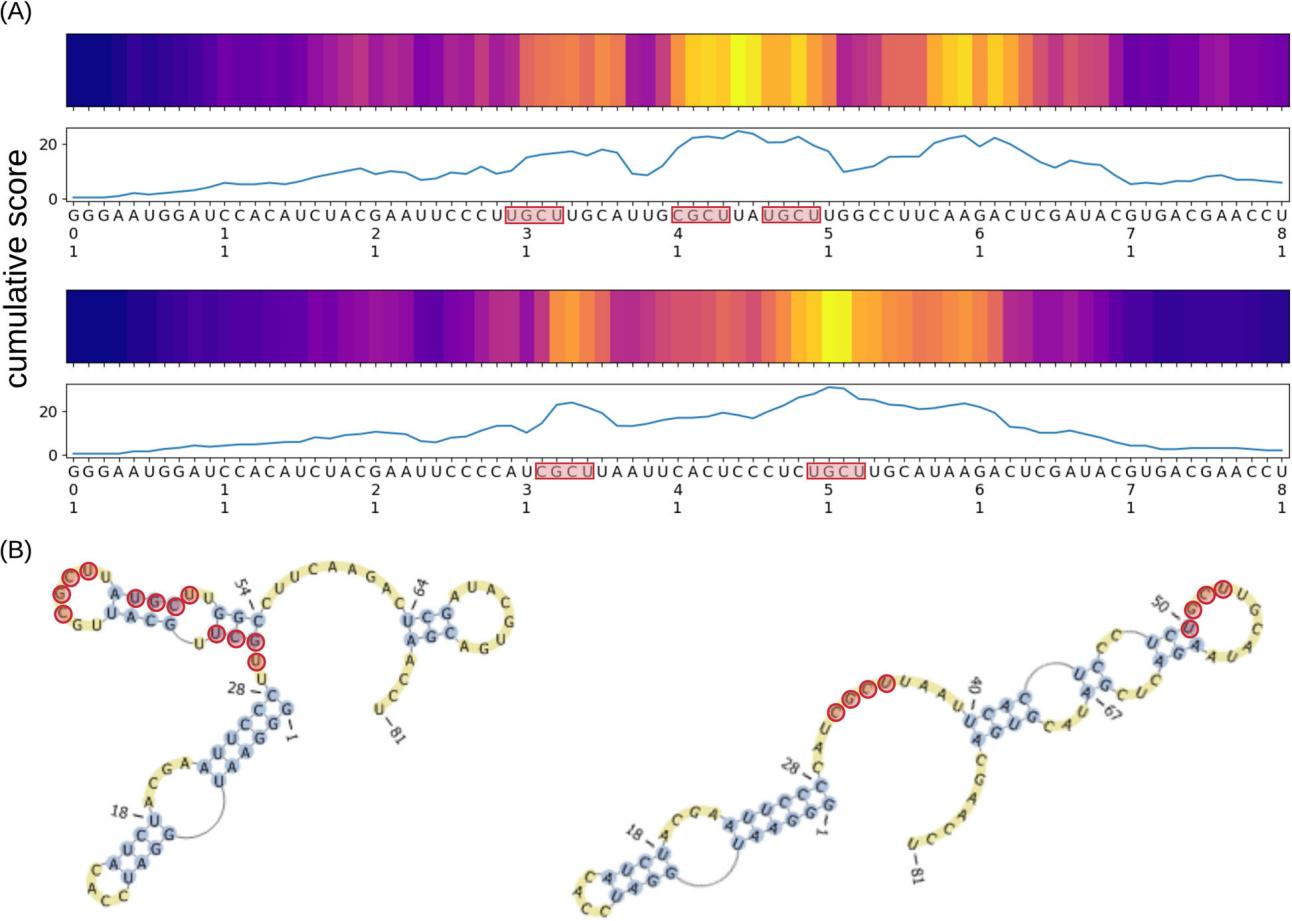
**a** The alignment of the generated RNA sequences to the known MBNL1-binding aptamer [28]. **b** Secondary structures of the known MBNL1-binding aptamers, visualized by PseudoViewer [29]. YGCY motifs are shown in red, where Y denotes pyrimidine (C or U)

In both alignments shown in Fig. 9, the highest cumulative score of the binding specificity was observed within the 32-mer MBNL1-binding regions. MBNL1-binding RNAs are known to contain YGCY motifs in their binding regions, where Y denotes pyrimidine (C or U) [28]. It is interesting to note that the motif is observed 3 times (positions 30–33, 41–44 and 47–50) in the 32-mer region of the first alignment, and twice (positions 32–35 and 50–53) in the second alignment of Fig. 9. Our model for RNA sequences was trained on data from in vivo experiments (i.e., CLIP-seq), yet generated RNA sequences with similar binding properties as those found by in vitro experiments (i.e., SELEX).

### Comparison with other methods

In “Binding affinity of generated sequences” section and Fig. 5, we compared the binding affinity of the DNA sequences generated by our model with the binding affinity of the DNA sequences generated by AptaSim and random generators. For more extensive comparison, we downloaded HT-SELEX fastq files of NFATC1 and NFKB1 from SRA (https://www.ncbi.nlm.nih.gov/bioproject/371436), and ran AptaSim. We selected the sequences after 10 rounds and generated motifs conserved in the sequences in the same way that we did for the motifs shown in Figs. 6 and 7. As shown in Additional file 5, the binding motifs conserved in the sequences generated by AptaSim are very different from the well-known binding motifs for NFATC1 (Fig. 6) and NFKB1 (Fig. 7).

For further comparison, we tried a different set of programs in the AptaSuite collection. We first generated a pool of DNA sequences with AptaSim, clustered the sequences with AptaCluster, and found 6- to 10-mer motifs with AptaTRACE. Several motifs were found in the sequences, but the well-known motifs (the NFATC1-binding motif TTTCCA and the NFKB1-binding motif GGGGGAATCCCC) were not included in the motifs. Details of the results are available in Additional file 5.

## Conclusion

Many studies have investigated the interactions between nucleic acids and proteins by computational approaches. However, most of the computational approaches treat the problem of nucleic acid-protein interactions as a classification problem. In this paper we proposed a generative model using a recurrent neural network (RNN) to generate nucleic acid sequences binding to a target protein. The model was trained on a huge set of sequences from high-throughput experimental technologies, and tested to construct nucleic acid sequences binding to a target protein. Both DNA and RNA sequences generated by the model for several target proteins showed a high binding specificity, and motifs observed in the sequences were similar to known motifs.

These are preliminary results of ongoing research, but demonstrated the potential of our approach as a generator of nucleic acid sequences binding to a target protein. In particular, our model will be useful in substantially reducing time and money for in vitro selection of aptamers such as SELEX experiments by constructing an efficient initial pool of nucleic acid sequences.

## Supplementary information

**Additional file 1** HT-SELEX data of DeepBind. The data used for training our generator model and the HT-SELEX data used for comparing with other methods. The size of the compressed file is about 1.8GB.

**Additional file 2** Nucleic acid sequences of three types. Nucleic acid sequences generated by our model, AptaSim, and random generator for four proteins (DRGX, GCM1, OLIG1 and RXRB).

**Additional file 3** Nucleic acid sequences generated by our model for three proteins. Nucleic acid sequences and their binding specificity to target proteins (NFATC1, NFKB1 and MBNL1), constructed by our model.

**Additional file 4** Known aptamers binding to three proteins. An aptamer binding to NFATC1, two aptamers binding to NFKB1, and two aptamers binding to MBNL1.

**Additional file 5** FATC1-binding motifs and NFKB1-binding motifs found in the DNA sequences generated by other methods. NFATC1-binding motifs and NFKB1-binding motifs found in the DNA sequences generated by AptaSim and by a set of programs in AptaSuite.

## Data Availability

Additional files are available at http://bclab.inha.ac.kr/aptamer.

## Abbreviations

AF: Binding affinity
IU: Intersection to union
LSTM: Long short-term memory
RNN: Recurrent neural network
SP: Binding specificity
