# Supplementary material for: A generative model for constructing nucleic acid sequences binding to a protein
Source: BMC Genomics. 2019 Dec 27;20(Suppl 13):967. doi: 10.1186/s12864-019-6299-4 (PMC6933682; doi:10.1186/s12864-019-6299-4)

ID	Motif Profile	Seed	Seed P-value	Seed Freq.	Motif Freq.	K-context Trace
1)		ATACAG	3.416E-3	5.24%	5.64%	
2)		AAATAC	2.293E-4	5.07%	6.92%	
3)		CGAGTG	9.175E-3	4.50%	6.96%	
4)		CCAGCT	4.735E-3	4.15%	5.91%	
5)		AGATTT	1.198E-4	3.97%	9.20%	
6)		ACCCAC	9.182E-3	3.93%	4.42%	
7)		CACCTG	1.126E-3	3.70%	9.04%	
8)		CCTTAC	6.758E-4	3.22%	8.70%	
9)		GAGCCG	3.745E-3	3.09%	3.24%	
10)		AGTGTC	2.833E-3	2.90%	3.01%	
11)		GATAAA	7.527E-3	2.77%	2.86%	
12)		GGATAT	6.601E-4	2.64%	4.02%	
13)		GTGGAA	9.187E-4	2.58%	6.90%	
14)		ATATCT	4.383E-4	2.46%	3.23%	
15)		TTAGAG	3.327E-5	2.42%	4.19%	
16)		GACATT	7.888E-4	2.30%	5.56%	
17)		GAAGCC	1.087E-5	2.09%	3.73%	
18)		AGAAGA	8.194E-5	1.93%	3.84%	
19)		GTAAGA	2.105E-4	1.78%	1.99%	
20)		GGTGAG	3.769E-3	1.66%	2.55%	
21)		AGCCAG	8.881E-3	1.60%	2.25%	
22)		TTTTTG	1.125E-3	1.40%	1.51%	
23)		GTCGAG	2.591E-4	1.32%	1.59%	
24)		CGGCGC	6.461E-4	1.06%	1.09%	
25)		AATTCG	1.058E-3	1.06%	1.10%	

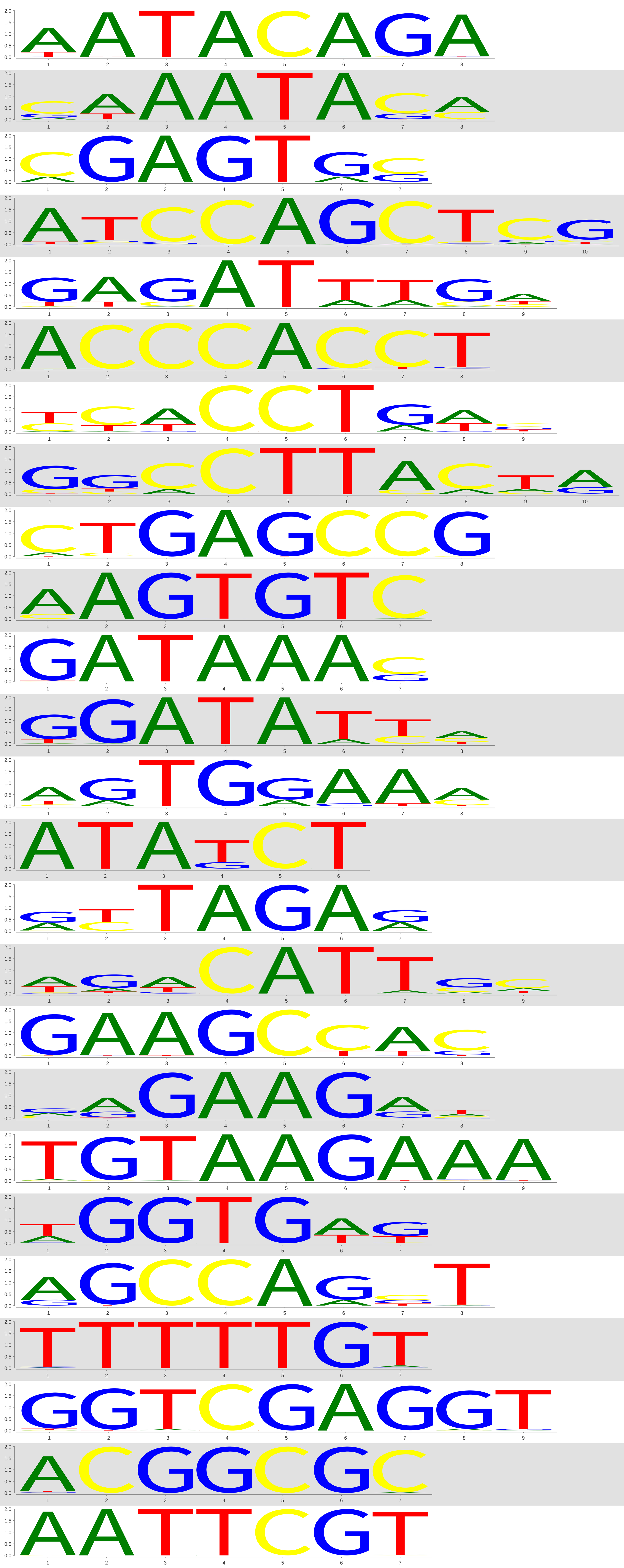

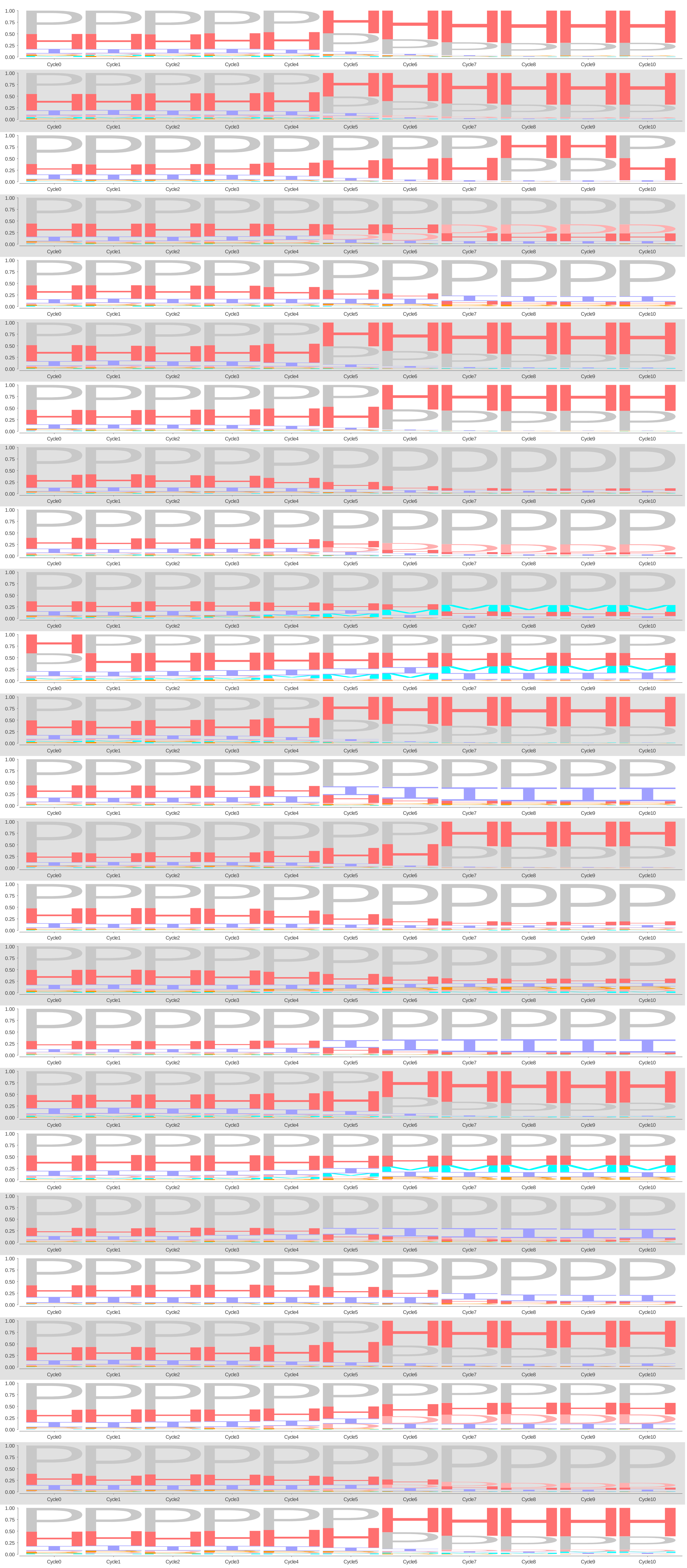

Supplement: Supplementary file 5 — Additional file 5 FATC1-binding motifs and NFKB1-binding motifs found in the DNA sequences generated by other methods. NFATC1-binding motifs and NFKB1-binding motifs found in the DNA sequences generated by AptaSim and by a set of programs in AptaSuite. [file 12864_2019_6299_MOESM5_ESM.zip › Additional_FIle_5/AptaTRACE/NFKB1/k6alpha10.pdf]
