# Supplementary material for: A generative model for constructing nucleic acid sequences binding to a protein
Source: BMC Genomics. 2019 Dec 27;20(Suppl 13):967. doi: 10.1186/s12864-019-6299-4 (PMC6933682; doi:10.1186/s12864-019-6299-4)
Supplement: Supplementary file 5 — Additional file 5 FATC1-binding motifs and NFKB1-binding motifs found in the DNA sequences generated by other methods. NFATC1-binding motifs and NFKB1-binding motifs found in the DNA sequences generated by AptaSim and by a set of programs in AptaSuite. [file 12864_2019_6299_MOESM5_ESM.zip › Additional_FIle_5/AptaTRACE/NFKB1/k9alpha10.pdf]

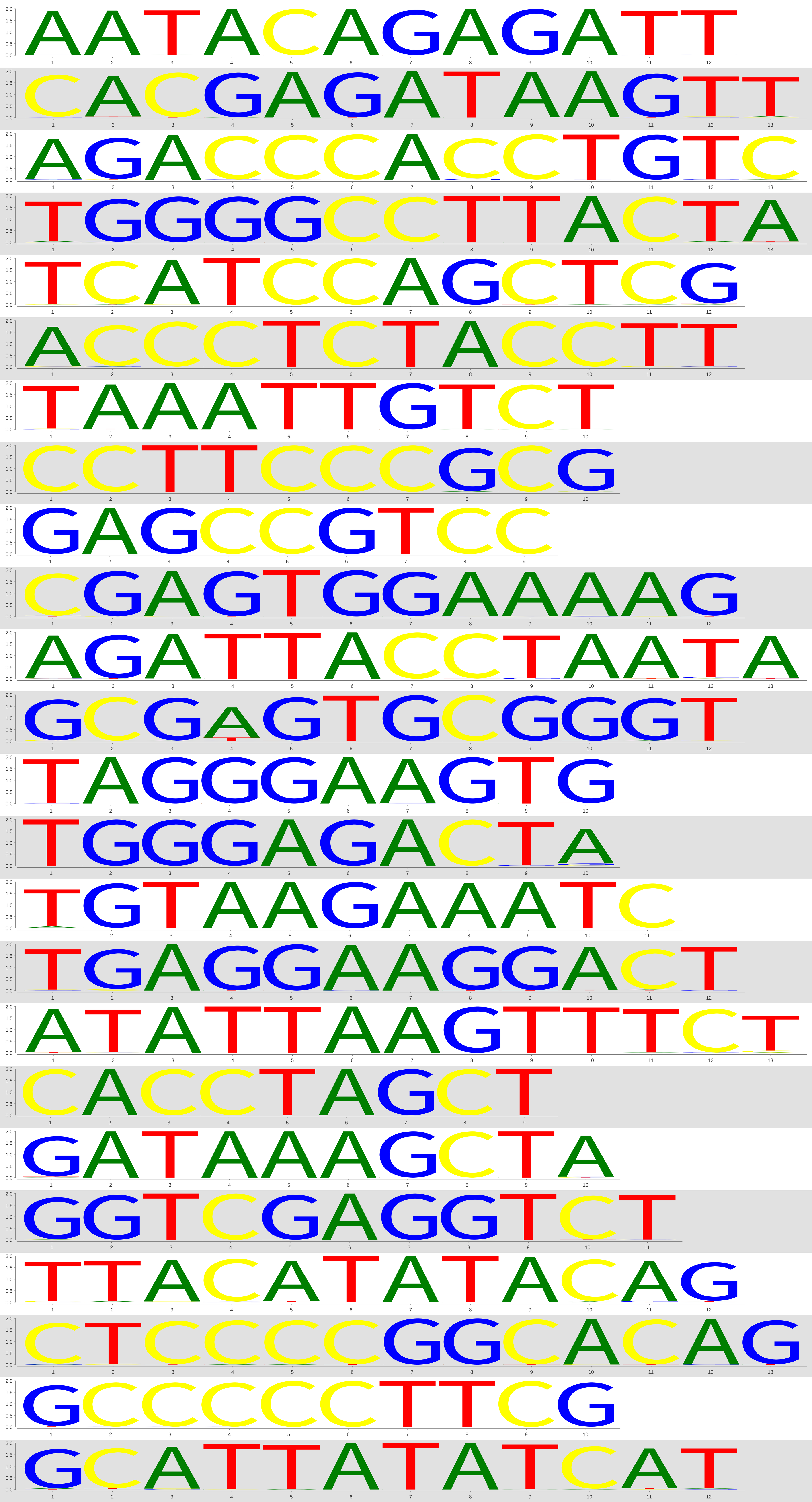

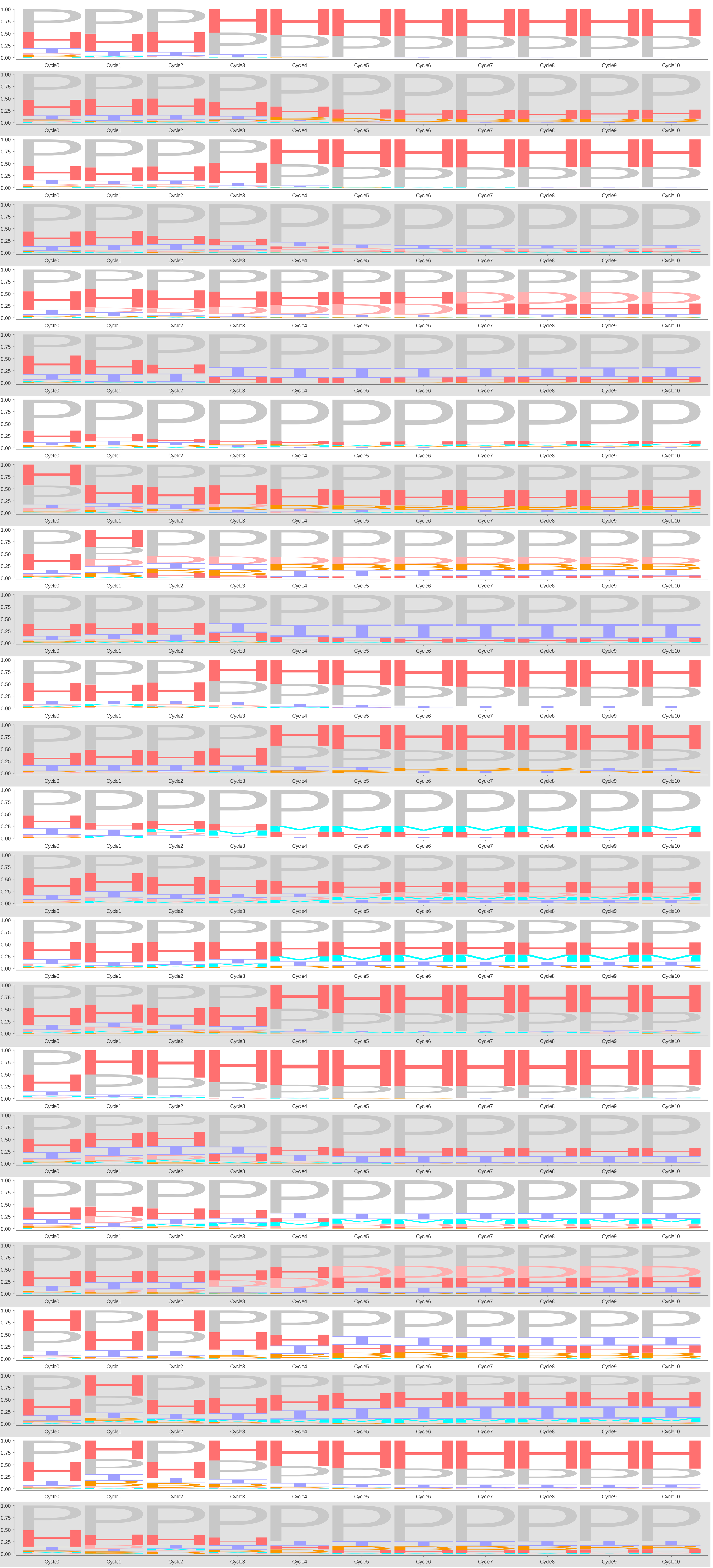

ID	Motif Profile	Seed	Seed P-value	Seed Freq.	Motif Freq.	K-context Trace
1)		ATACAGAGA	1.348E-13	3.74%	3.95%	
2)		CGAGATAAG	2.403E-19	3.39%	3.57%	
3)		ACCCACCTG	6.528E-20	3.14%	3.58%	
4)		GGGCCTTAC	7.088E-40	2.99%	3.00%	
5)		CATCCAGCT	2.051E-23	2.50%	2.65%	
6)		CCTCTACCT	1.28E-35	2.15%	2.30%	
7)		AAATTGTCT	3.474E-14	2.15%	2.17%	
8)		CCTTCCCGC	9.136E-19	2.03%	2.06%	
9)		GAGCCGTCC	2.48E-36	2.02%	2.02%	
10)		GAGTGGAAA	8.872E-26	1.97%	2.00%	
11)		ATTACCTAA	5.135E-10	1.92%	2.20%	
12)		CGAGTGCGG	1.497E-18	1.88%	2.23%	
13)		AGGGAAGTG	2.446E-51	1.83%	1.90%	
14)		TGGGAGACT	2.429E-14	1.66%	1.68%	
15)		GTAAGAAAT	7.18E-22	1.57%	1.61%	
16)		GAGGAAGGA	2.912E-24	1.46%	1.53%	
17)		ATTAAGTTT	3.914E-16	1.38%	1.45%	
18)		CACCTAGCT	9.209E-9	1.35%	1.35%	
19)		GATAAAGCT	1.101E-27	1.15%	1.21%	
20)		GTCGAGGTC	1.969E-31	1.14%	1.22%	
21)		TACATATAC	6.237E-40	1.12%	1.20%	
22)		CCCCGGCAC	3.632E-13	1.12%	1.14%	
23)		CCCCCTTCG	4.872E-14	1.09%	1.11%	
24)		ATTATATCA	3.219E-25	1.05%	1.16%	
