# Supplementary material for: A generative model for constructing nucleic acid sequences binding to a protein
Source: BMC Genomics. 2019 Dec 27;20(Suppl 13):967. doi: 10.1186/s12864-019-6299-4 (PMC6933682; doi:10.1186/s12864-019-6299-4)
Supplement: Supplementary file 5 — Additional file 5 FATC1-binding motifs and NFKB1-binding motifs found in the DNA sequences generated by other methods. NFATC1-binding motifs and NFKB1-binding motifs found in the DNA sequences generated by AptaSim and by a set of programs in AptaSuite. [file 12864_2019_6299_MOESM5_ESM.zip › Additional_FIle_5/AptaTRACE/NFKB1/k7alpha10.pdf]

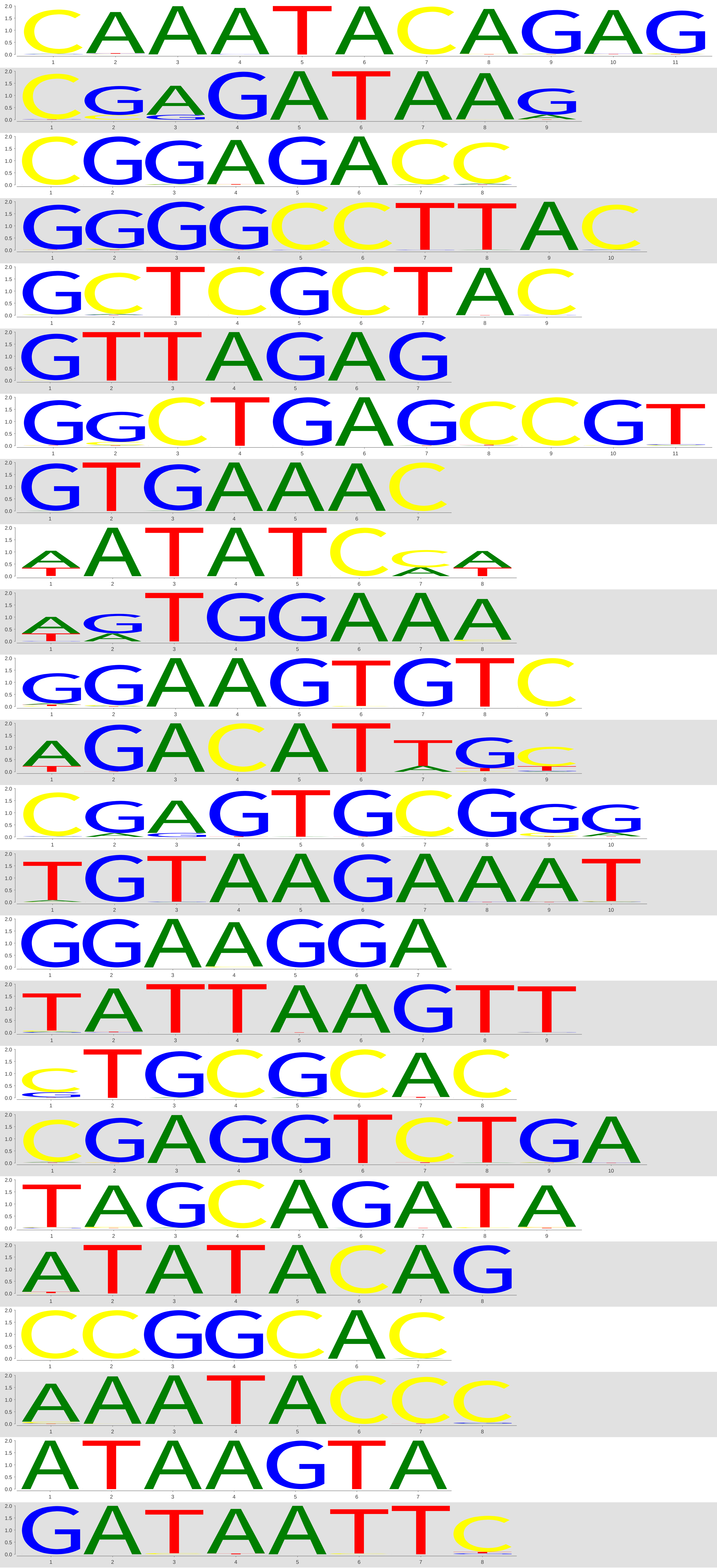

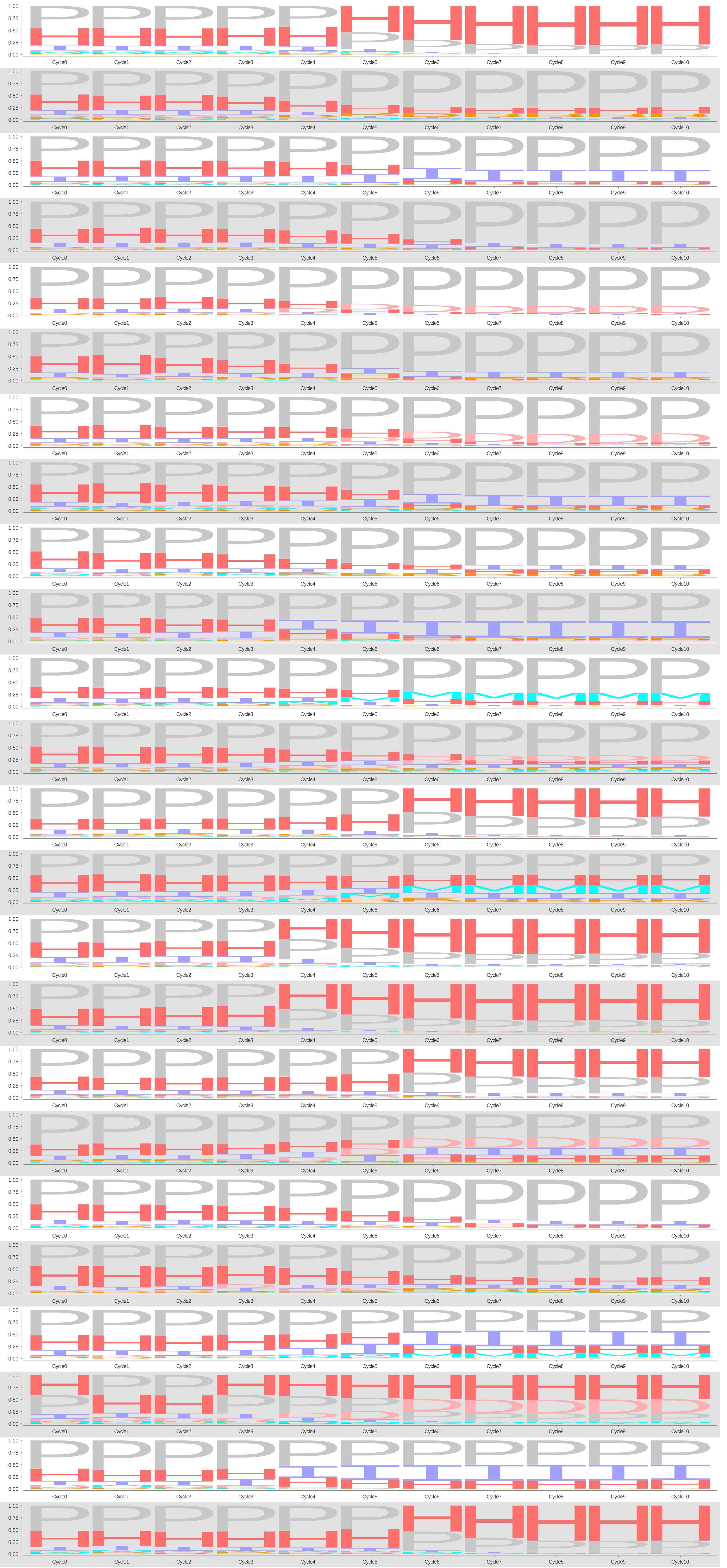

ID	Motif Profile	Seed	Seed P-value	Seed Freq.	Motif Freq.	K-context Trace
1)		AATACAG	2.252E-3	3.94%	4.28%	
2)		GAGATAA	4.256E-3	3.54%	4.88%	
3)		CGGAGAC	5.92E-3	3.45%	3.67%	
4)		GGCCTTA	3.748E-3	3.13%	3.30%	
5)		TCGCTAC	6.973E-3	2.68%	2.75%	
6)		GTTAGAG	1.734E-3	2.32%	2.34%	
7)		CTGAGCC	7.335E-3	2.19%	2.30%	
8)		GTGAAAC	1.775E-3	2.17%	2.22%	
9)		AATATCC	9.552E-3	2.13%	3.22%	
10)		GTGGAAA	6.319E-4	2.06%	3.08%	
11)		AAGTGTC	8.838E-3	2.05%	2.09%	
12)		AGACATT	7.529E-3	2.02%	2.56%	
13)		GAGTGCG	3.948E-3	1.97%	2.36%	
14)		GTAAGAA	1.088E-3	1.64%	1.80%	
15)		GGAAGGA	1.667E-3	1.54%	1.57%	
16)		TTAAGTT	7.764E-3	1.49%	1.58%	
17)		TGCGCAC	9.579E-3	1.47%	1.54%	
18)		AGGTCTG	1.43E-3	1.23%	1.39%	
19)		AGCAGAT	6.736E-3	1.23%	1.32%	
20)		TATACAG	9.541E-3	1.21%	1.21%	
21)		CCGGCAC	6.139E-3	1.20%	1.22%	
22)		AATACCC	1.636E-3	1.15%	1.25%	
23)		ATAAGTA	6.913E-3	1.04%	1.04%	
24)		GATAATT	5.979E-3	1.03%	1.12%	
