# Supplementary material for: A generative model for constructing nucleic acid sequences binding to a protein
Source: BMC Genomics. 2019 Dec 27;20(Suppl 13):967. doi: 10.1186/s12864-019-6299-4 (PMC6933682; doi:10.1186/s12864-019-6299-4)
Supplement: Supplementary file 5 — Additional file 5 FATC1-binding motifs and NFKB1-binding motifs found in the DNA sequences generated by other methods. NFATC1-binding motifs and NFKB1-binding motifs found in the DNA sequences generated by AptaSim and by a set of programs in AptaSuite. [file 12864_2019_6299_MOESM5_ESM.zip › Additional_FIle_5/AptaTRACE/NFKB1/k8alpha10.pdf]

ID	Motif Profile	Seed	Seed P-value	Seed Freq.	Motif Freq.	K-context Trace
1)	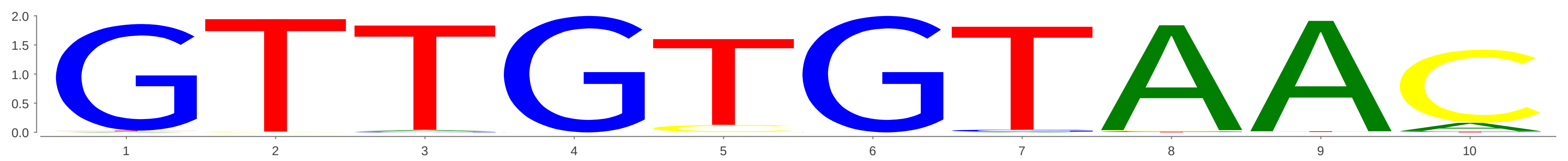	TTGTGTAA	3.645E-3	2.95%	3.57%	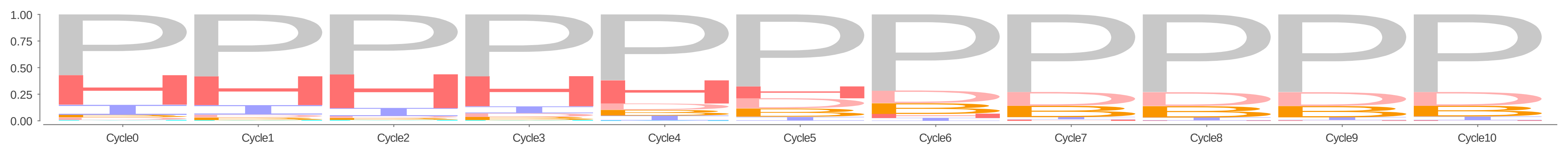
2)	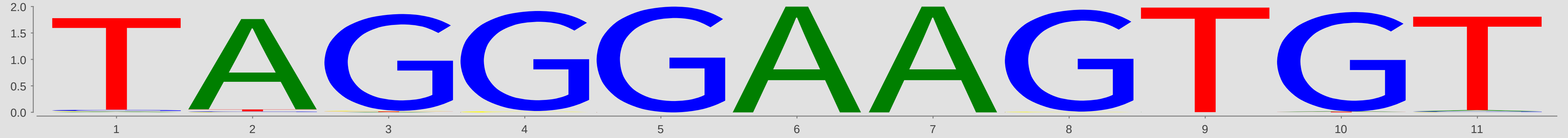	GGGAAGTG	5.745E-3	1.87%	2.04%	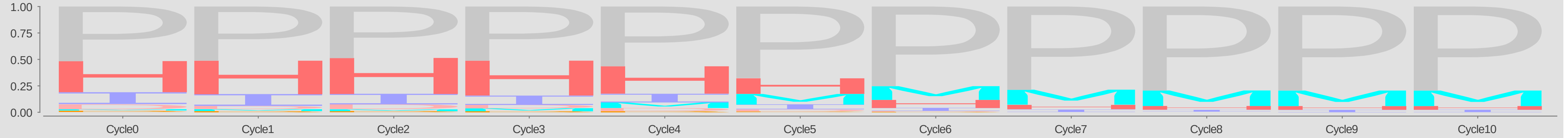
3)	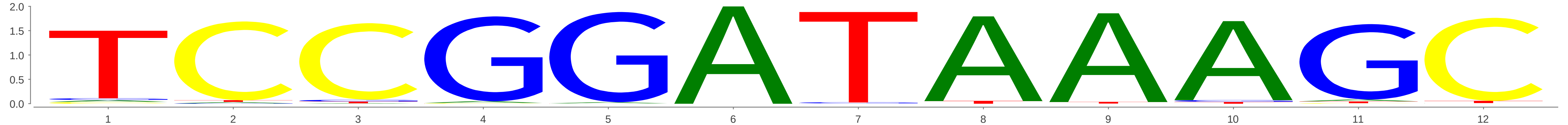	CGGATAAA	2.768E-3	1.17%	1.31%	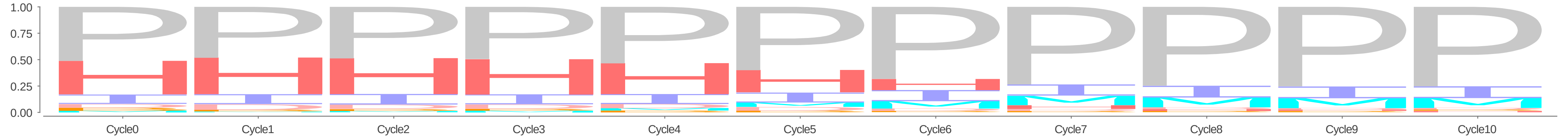
