# Supplementary material for: A generative model for constructing nucleic acid sequences binding to a protein
Source: BMC Genomics. 2019 Dec 27;20(Suppl 13):967. doi: 10.1186/s12864-019-6299-4 (PMC6933682; doi:10.1186/s12864-019-6299-4)
Supplement: Supplementary file 5 — Additional file 5 FATC1-binding motifs and NFKB1-binding motifs found in the DNA sequences generated by other methods. NFATC1-binding motifs and NFKB1-binding motifs found in the DNA sequences generated by AptaSim and by a set of programs in AptaSuite. [file 12864_2019_6299_MOESM5_ESM.zip › Additional_FIle_5/AptaTRACE/NFATC1/k6alpha10.pdf]

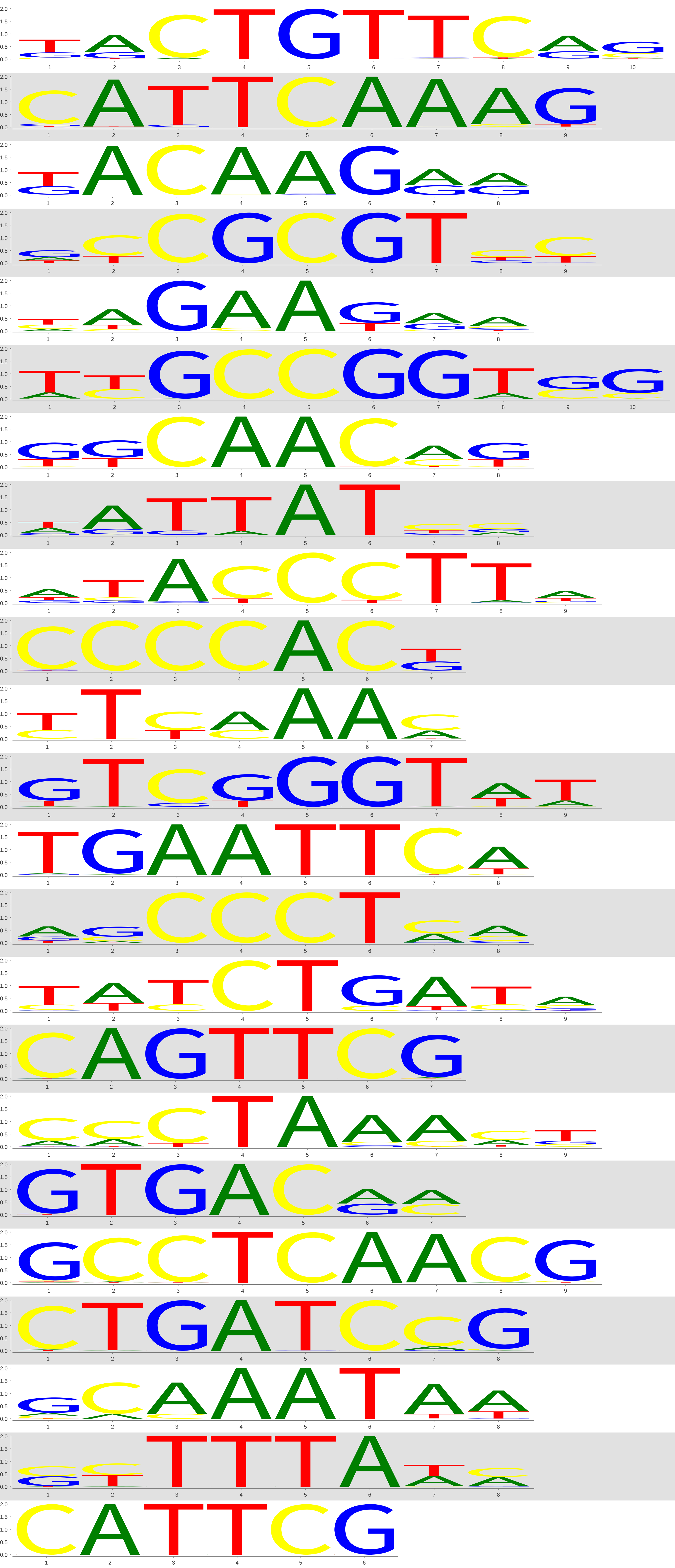

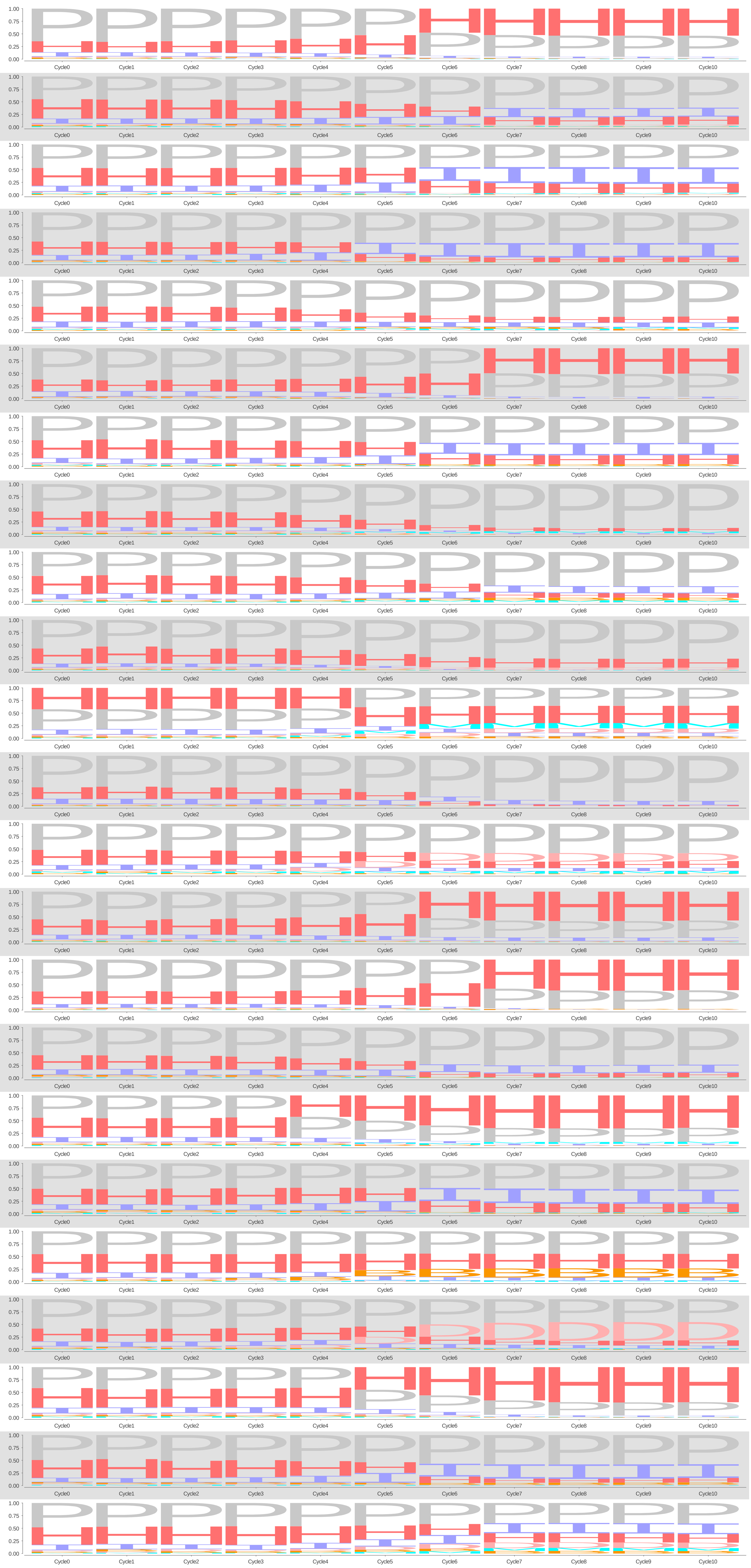

ID	Motif Profile	Seed	Seed P-value	Seed Freq.	Motif Freq.	K-context Trace
1)		CTGTTC	2.714E-3	8.51%	11.66%	
2)		ATTCAA	5.128E-3	4.71%	5.30%	
3)		ACAAGA	1.847E-3	4.58%	6.62%	
4)		CCGCGT	2.902E-3	4.56%	8.83%	
5)		CAGAAG	6.318E-3	4.29%	11.19%	
6)		GCCGGT	1.517E-3	4.25%	5.02%	
7)		GGCAAC	3.385E-3	4.16%	6.57%	
8)		ATTATC	1.865E-3	4.16%	6.56%	
9)		ACCCTT	7.747E-5	2.96%	5.27%	
10)		CCCCAC	4.163E-3	2.69%	2.83%	
11)		TCAAAC	9.334E-3	2.36%	2.71%	
12)		TCGGGT	1.1E-3	2.27%	5.15%	
13)		TGAATT	9.511E-3	2.21%	2.47%	
14)		AGCCCT	5.062E-3	2.16%	3.60%	
15)		ATCTGA	1.011E-4	1.87%	3.44%	
16)		CAGTTC	5.773E-3	1.71%	1.79%	
17)		CCTAAA	7.102E-3	1.65%	4.06%	
18)		TGACAA	3.622E-3	1.53%	2.73%	
19)		CCTCAA	7.055E-4	1.34%	1.59%	
20)		CTGATC	4.137E-5	1.24%	1.36%	
21)		CAAATA	3.445E-4	1.17%	2.04%	
22)		CCTTTA	4.479E-5	1.05%	2.18%	
23)		CATTCG	1.603E-3	1.04%	1.04%	
