# Supplementary material for: A generative model for constructing nucleic acid sequences binding to a protein
Source: BMC Genomics. 2019 Dec 27;20(Suppl 13):967. doi: 10.1186/s12864-019-6299-4 (PMC6933682; doi:10.1186/s12864-019-6299-4)
Supplement: Supplementary file 5 — Additional file 5 FATC1-binding motifs and NFKB1-binding motifs found in the DNA sequences generated by other methods. NFATC1-binding motifs and NFKB1-binding motifs found in the DNA sequences generated by AptaSim and by a set of programs in AptaSuite. [file 12864_2019_6299_MOESM5_ESM.zip › Additional_FIle_5/AptaTRACE/NFATC1/k7alpha10.pdf]

ID	Motif Profile	Seed	Seed P-value	Seed Freq.	Motif Freq.	K-context Trace
1)	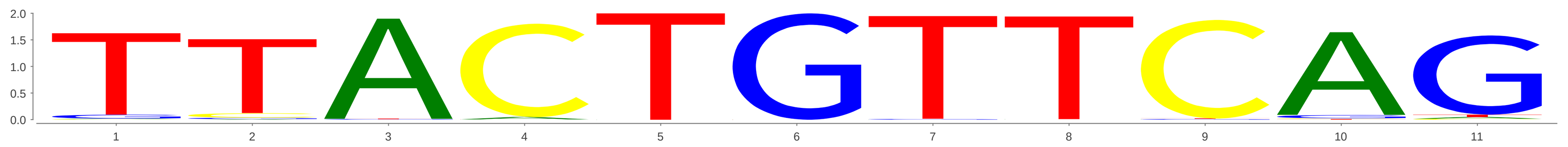	ACTGTTC	7.701E-3	4.79%	5.90%	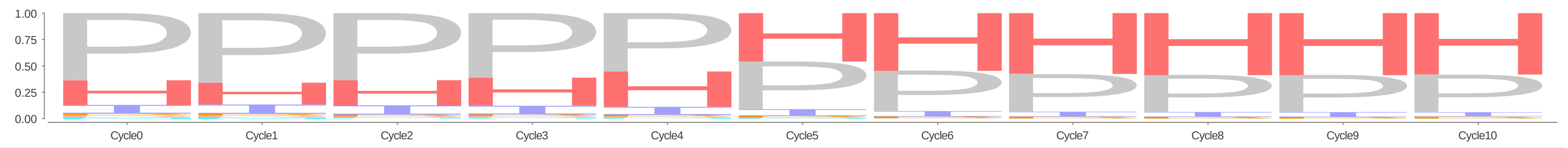
2)	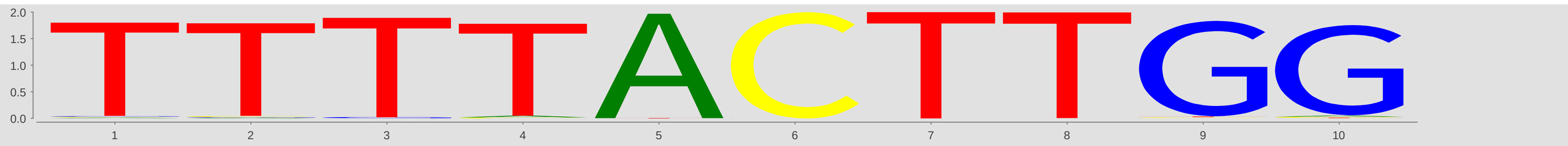	TTACTTG	5.782E-3	3.90%	4.38%	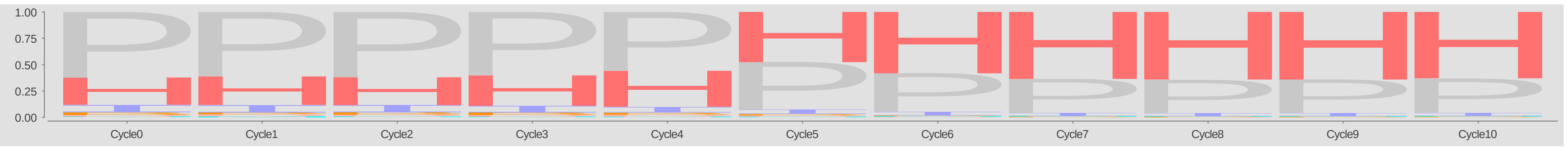
3)	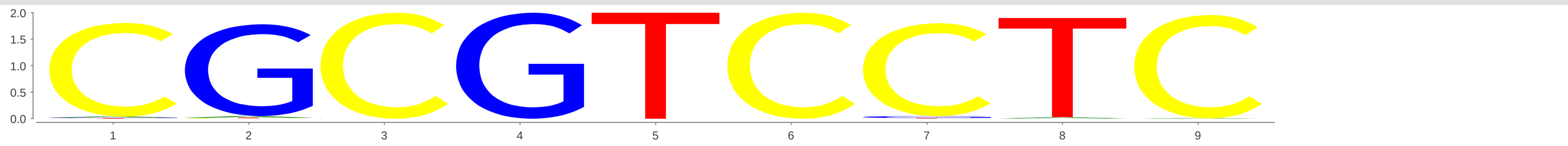	CGTCCTC	6.169E-3	2.71%	2.85%	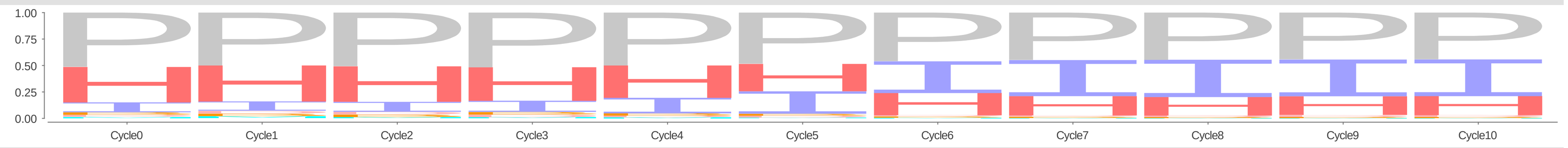
4)	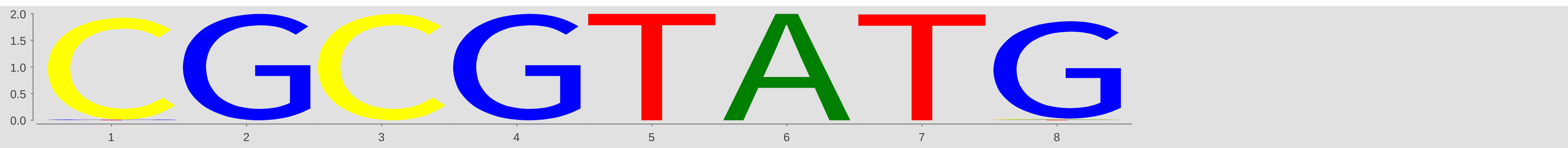	CGCGTAT	2.425E-3	2.18%	2.23%	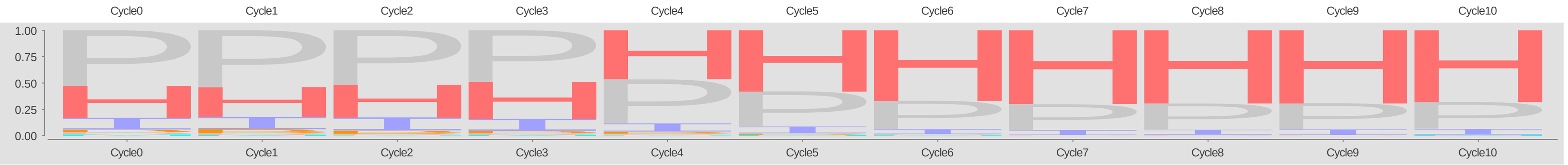
5)	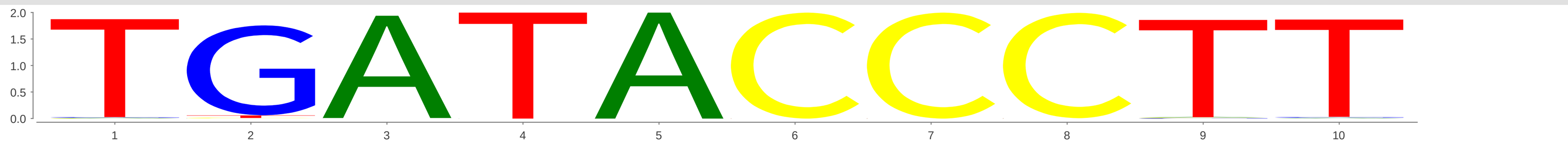	ATACCCT	9.539E-4	2.14%	2.27%	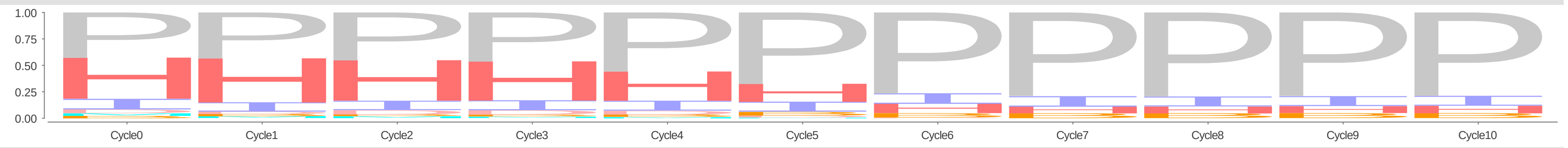
6)	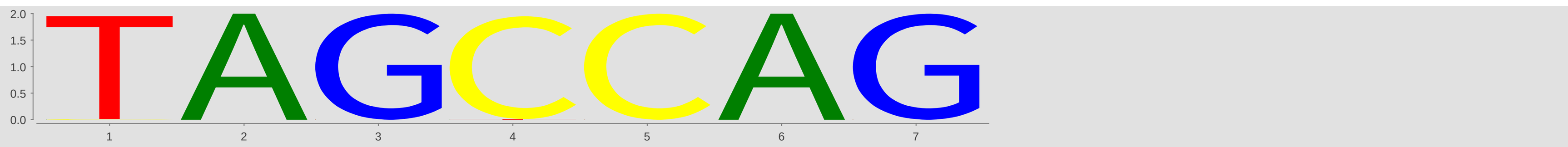	TAGCCAG	5.035E-4	1.91%	1.94%	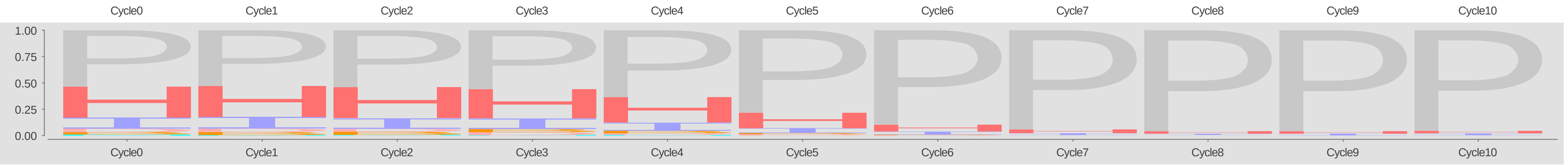
7)	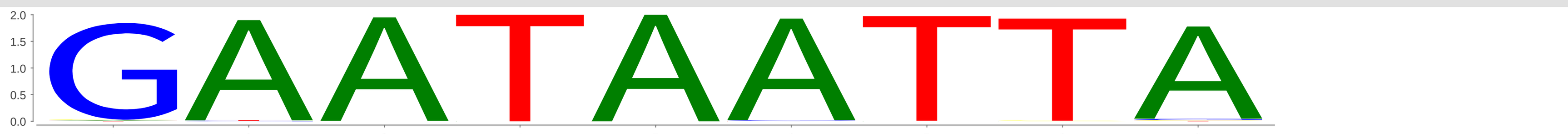	AATAATT	2.232E-3	1.85%	2.07%	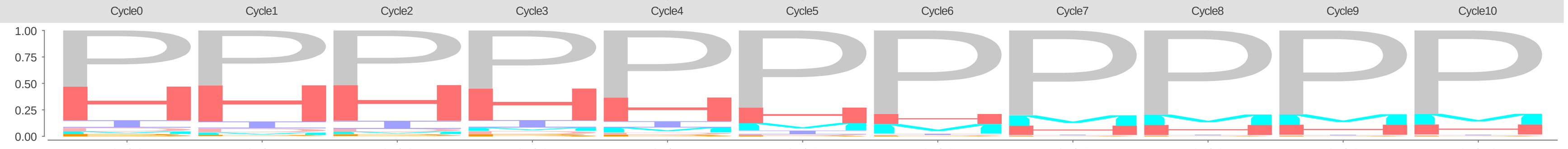
8)	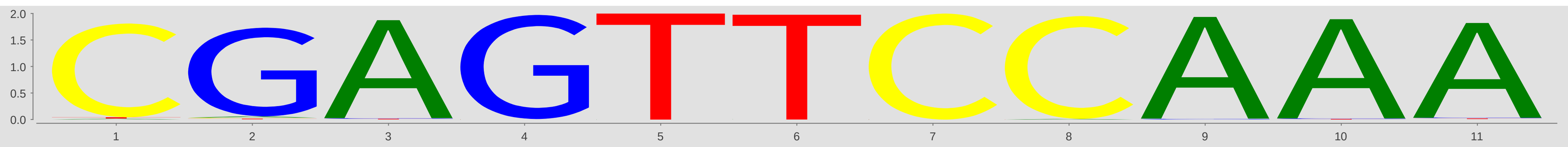	AGTTCCA	2.423E-3	1.83%	1.96%	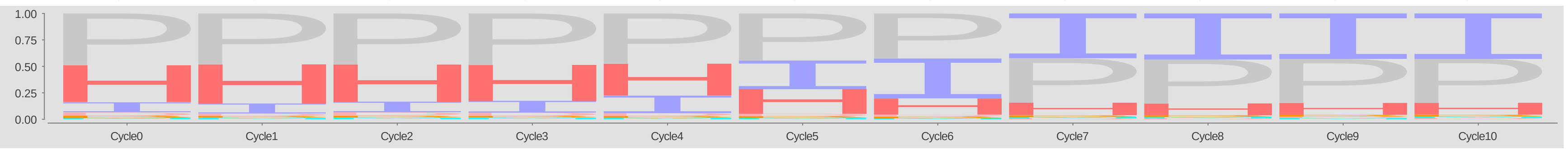
9)	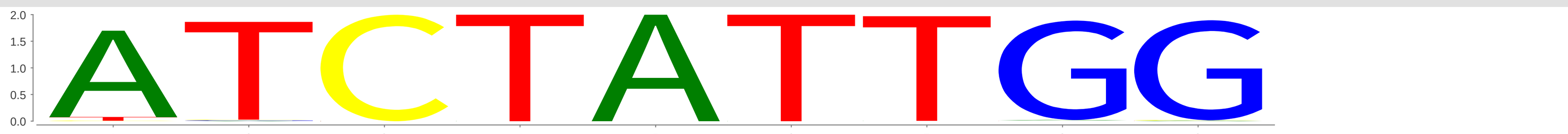	CTATTGG	7.689E-4	1.63%	1.72%	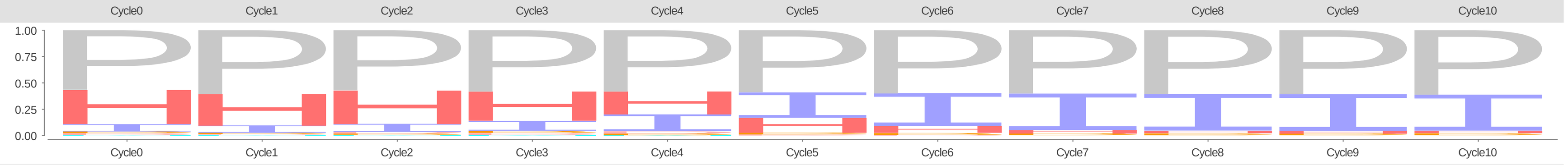
10)	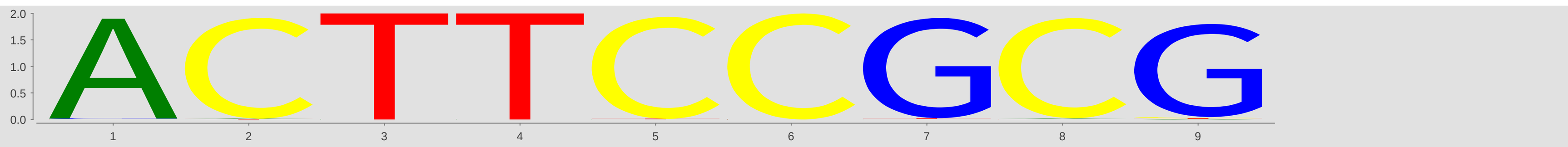	ACTTCCG	5.478E-4	1.57%	1.75%	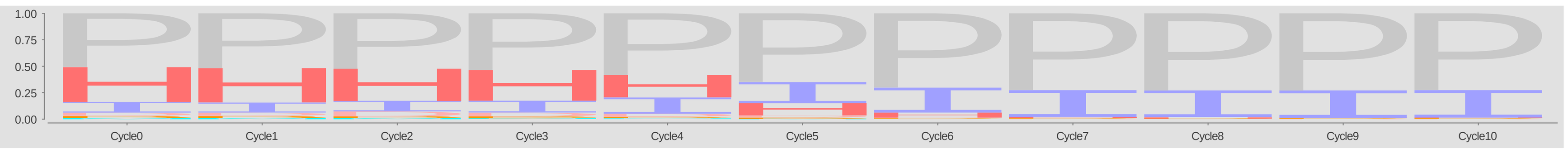
11)	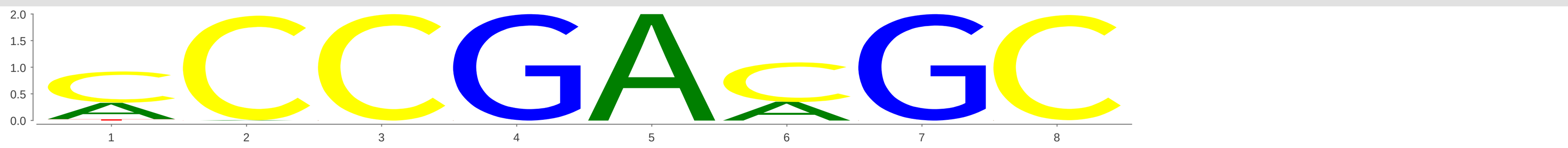	CCGACGC	6.045E-3	1.57%	2.38%	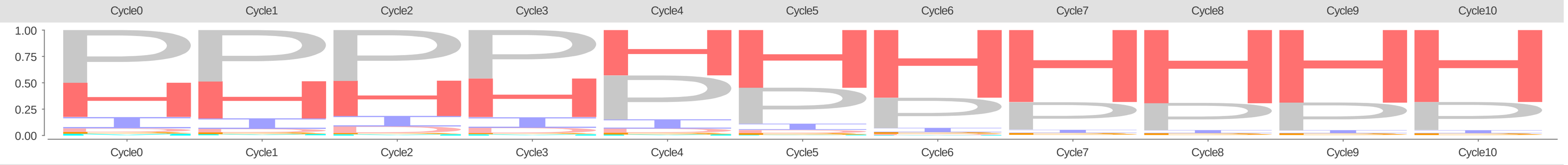
12)	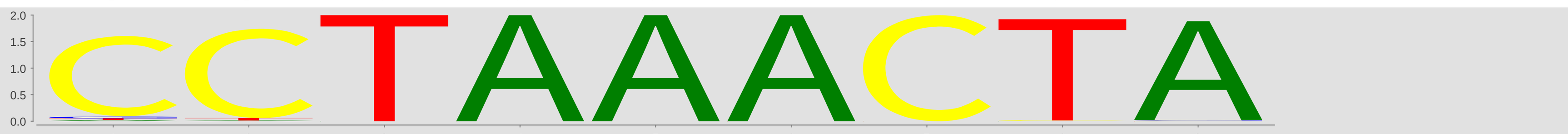	TAAACTA	8.926E-3	1.56%	1.61%	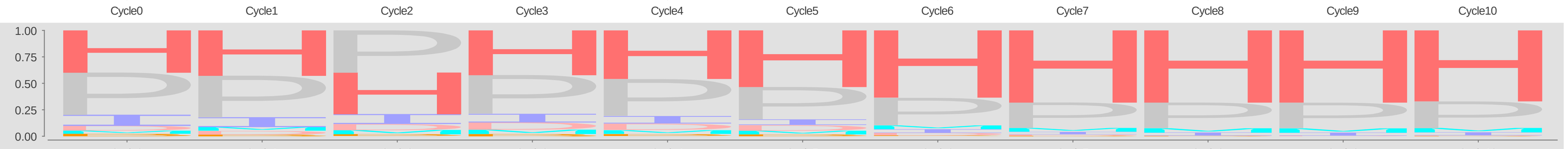
13)	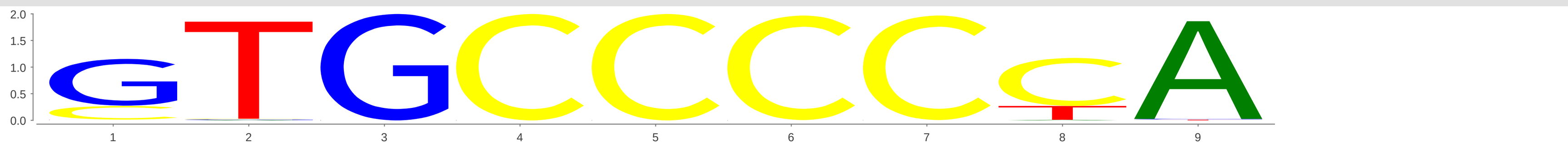	TGCCCCC	4.765E-3	1.52%	2.46%	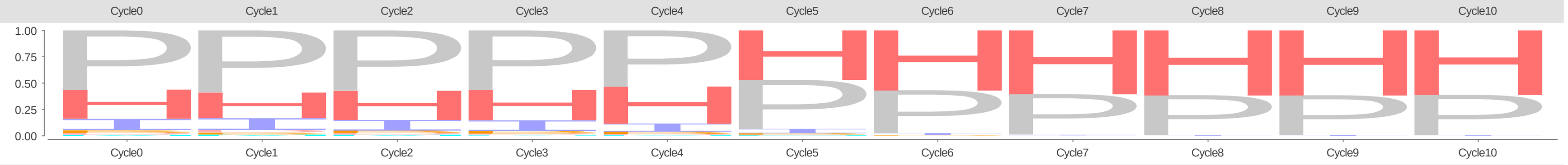
14)	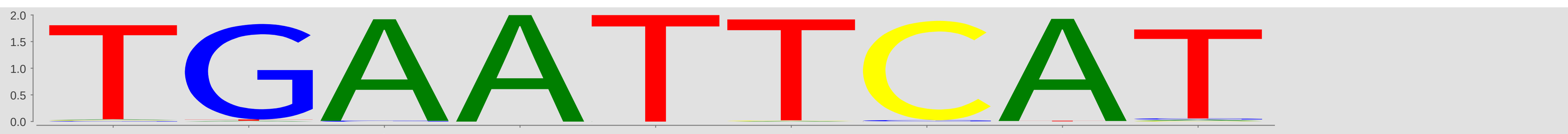	GAATTCA	3.508E-3	1.50%	1.59%	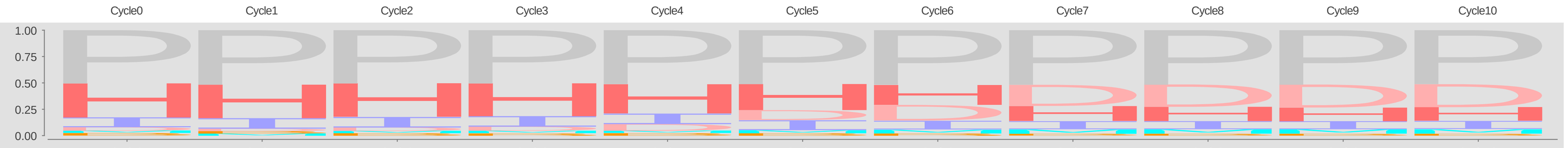
15)	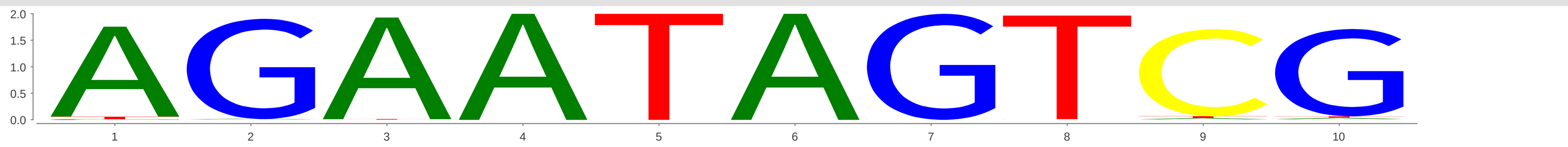	GAATAGT	8.497E-3	1.43%	1.54%	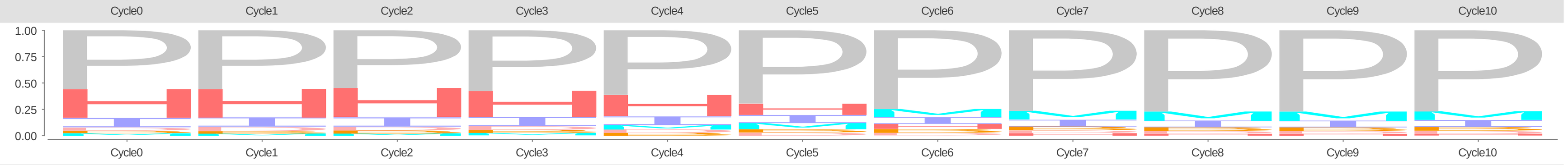
16)	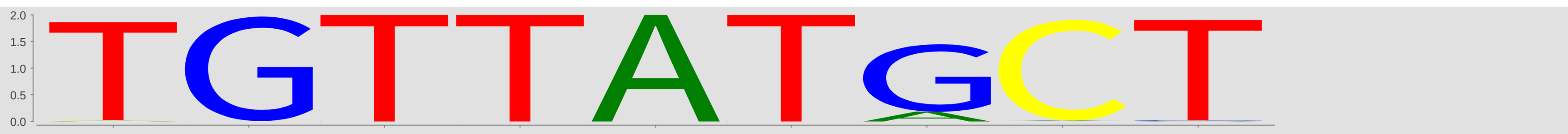	TGTTATG	2.79E-3	1.38%	1.76%	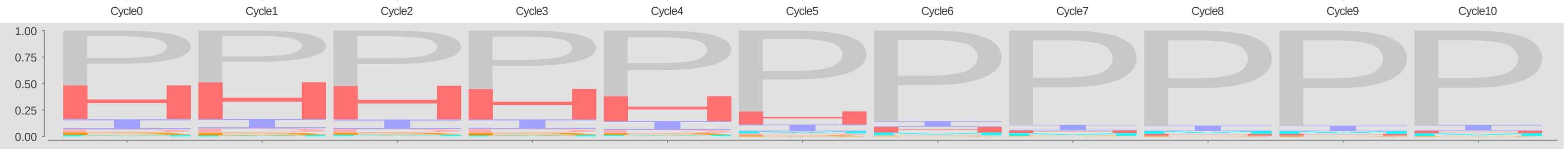
17)	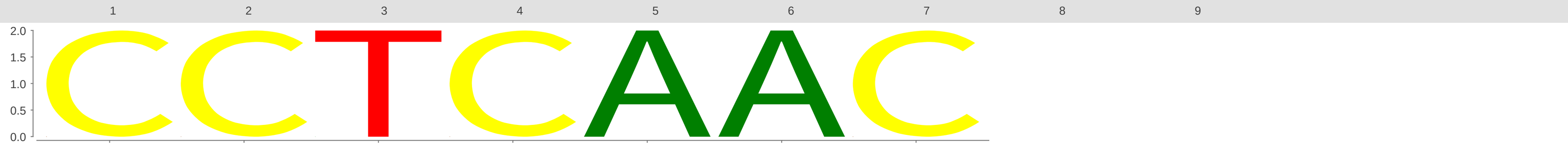	CCTCAAC	5.354E-3	1.23%	1.23%	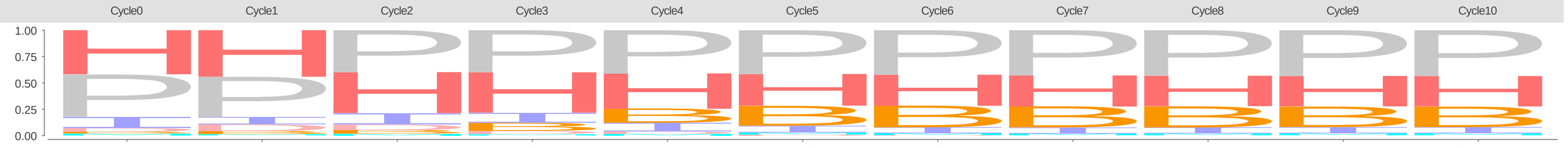
18)	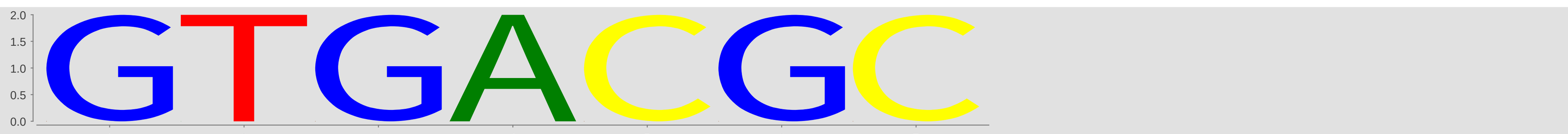	GTGACGC	4.905E-3	1.15%	1.15%	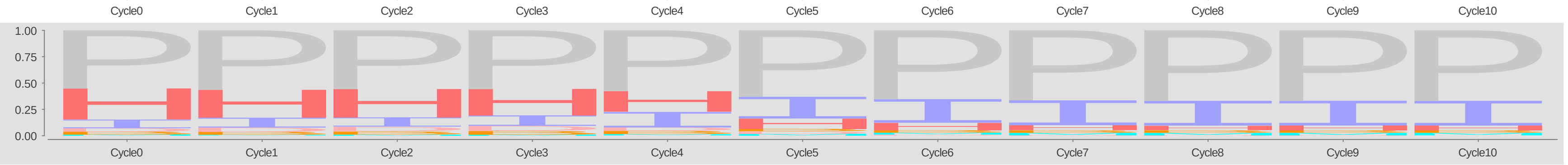
19)	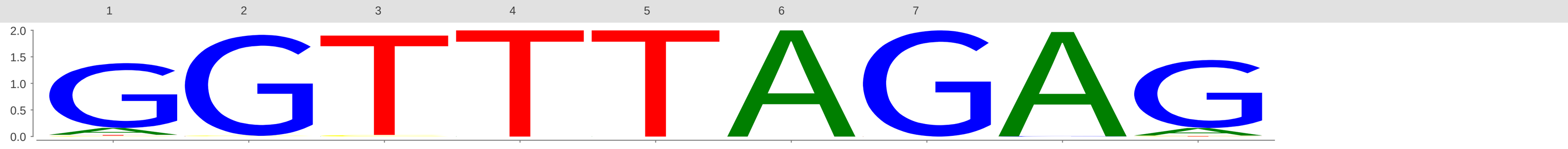	GTTTAGA	1.096E-3	1.12%	1.17%	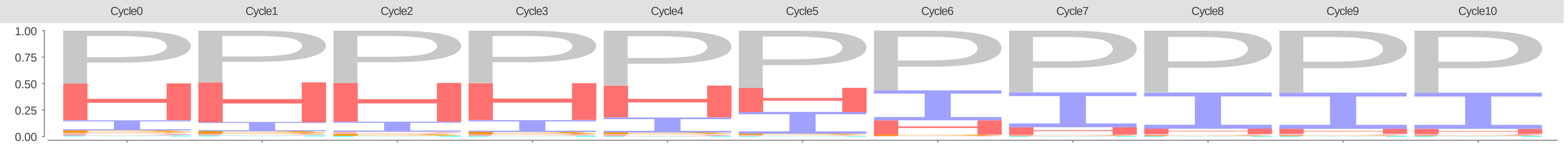
20)	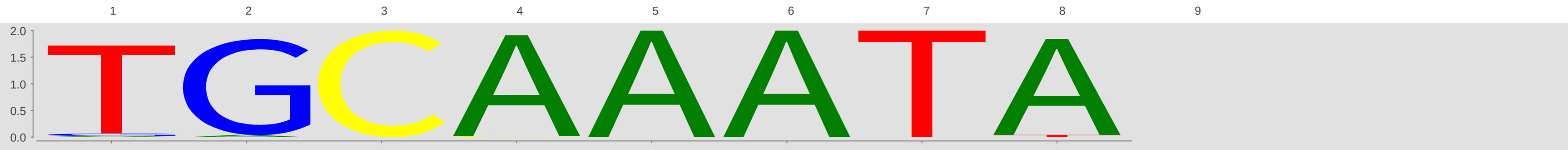	GCAAATA	6.262E-3	1.11%	1.16%	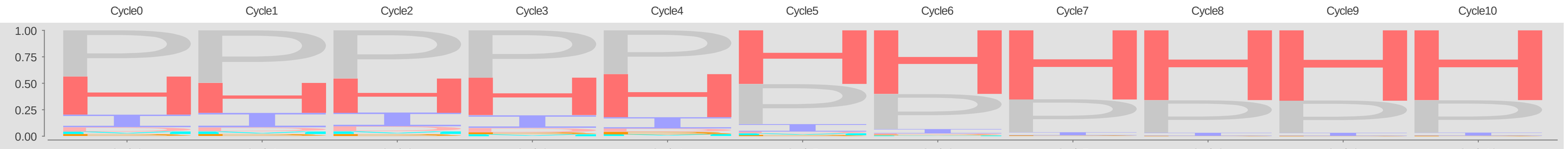
