# Supplementary material for: A generative model for constructing nucleic acid sequences binding to a protein
Source: BMC Genomics. 2019 Dec 27;20(Suppl 13):967. doi: 10.1186/s12864-019-6299-4 (PMC6933682; doi:10.1186/s12864-019-6299-4)
Supplement: Supplementary file 5 — Additional file 5 FATC1-binding motifs and NFKB1-binding motifs found in the DNA sequences generated by other methods. NFATC1-binding motifs and NFKB1-binding motifs found in the DNA sequences generated by AptaSim and by a set of programs in AptaSuite. [file 12864_2019_6299_MOESM5_ESM.zip › Additional_FIle_5/AptaTRACE/NFATC1/k8alpha10.pdf]

ID	Motif Profile	Seed	Seed P-value	Seed Freq.	Motif Freq.	K-context Trace
1)	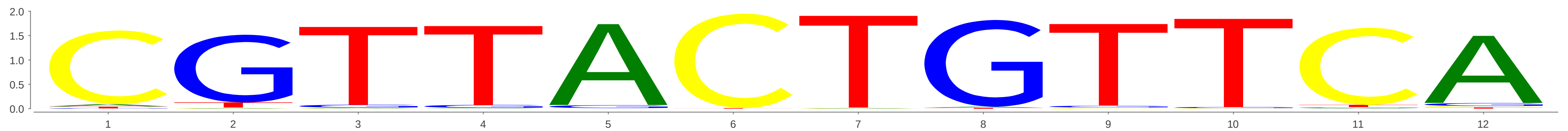	TTACTGTT	8.045E-3	3.94%	4.75%	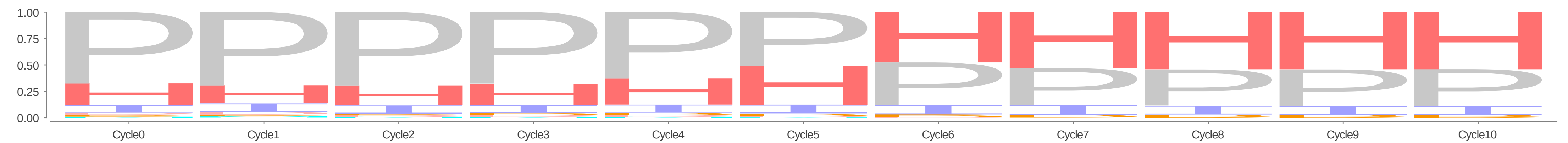
2)	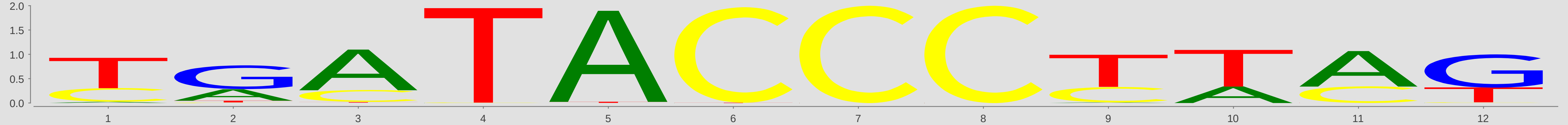	ATACCCTT	6.44E-3	2.06%	3.24%	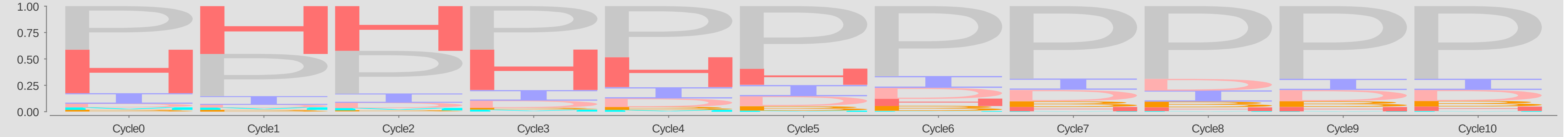
3)	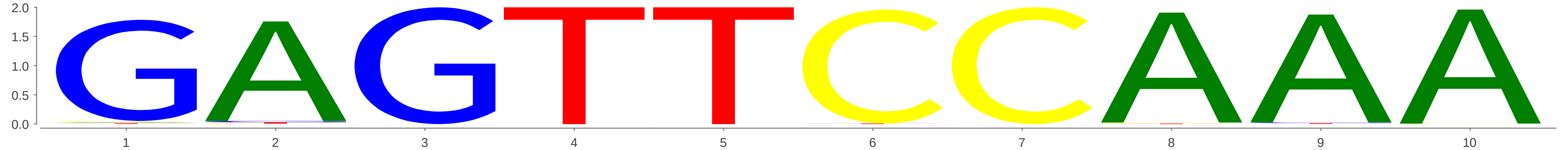	GTTCCAAA	5.781E-3	1.79%	1.86%	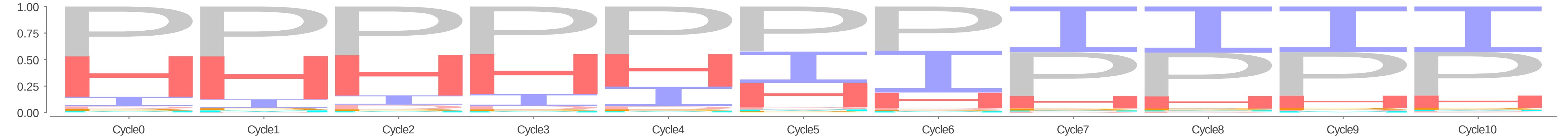
4)	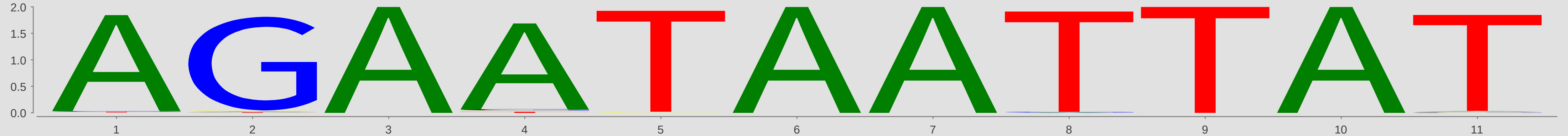	AATAATTA	9.407E-3	1.77%	1.85%	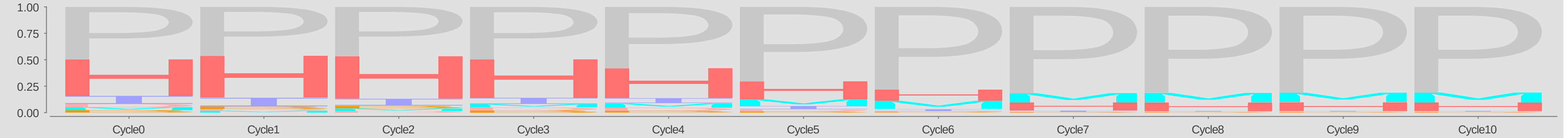
5)	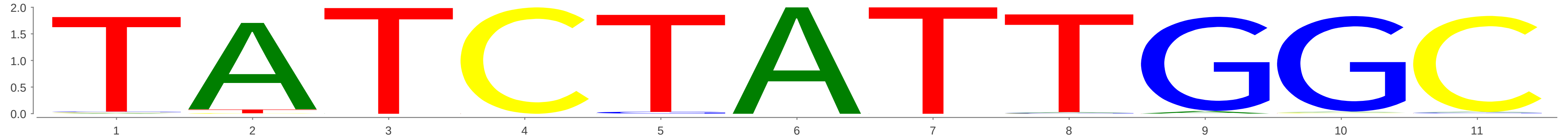	ATCTATTG	4.302E-3	1.50%	1.67%	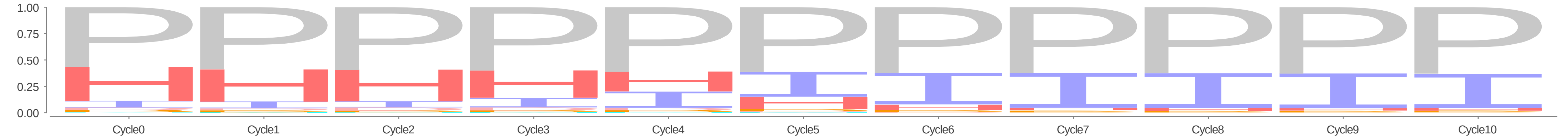
